# Design, Validation, and Metrological Limits of Biofidelic Instrumentation in PFL Collaborative Robotics: A Systematic Review of Longitudinal Trends and Future Paradigms

**DOI:** 10.3390/s26133984

**Published:** 2026-06-23

**Authors:** Daniel Hartmann, Kristýna Hamříková, Aleš Vysocký, Vendula Laciok, Aleš Bernatík

**Affiliations:** 1 Faculty of Mechanical Engineering, VSB—Technical University of Ostrava, 708 00 Ostrava, Czech Republic; ales.vysocky@vsb.cz; 2 Faculty of Safety Engineering, VSB—Technical University of Ostrava, 708 00 Ostrava, Czech Republic; kristyna.hamrikova@vsb.cz (K.H.); vendula.laciok@vsb.cz (V.L.); ales.bernatik@vsb.cz (A.B.)

**Keywords:** power and force limiting, physical human–robot interaction, biofidelic sensors, pressure and force measurement device, safety validation, metrology, collision dynamics, ISO 10218-2, ISO/TS 15066

## Abstract

The integration of collaborative robots into industrial environments requires rigorous safety validation under the Power and Force Limiting (PFL) regime. This review article systematically maps the technological and normative development of certified Pressure and Force Measurement Devices (PFMDs) and experimental biofidelic instruments for Physical Human–Robot Interaction (pHRI) between the years 2011 and 2026. A quantitative screening of 68 studies revealed a publication peak in impact metrology in 2021. This peak occurred with a five-year latency after the release of the ISO/TS 15066 technical specification. Although global interest in collaborative robotics steadily grows, the publication trend indicates a gradual shift in scientific focus from reactive testing toward proactive prevention. A methodological deconstruction of four Research Questions (RQs) identifies persistent limitations in safety evaluation. The findings demonstrate that the internal structure of conventional sensors induces nonlinear shock filtering and parasitic oscillations (RQ1). Furthermore, the rigid fixation of test stands generates unrealistic pressure spikes. This physical limitation forces a transition to flexible and pendulum-based configurations (RQ2). Commercial flat films physically fail due to sensor saturation and introduced stiffness. Such failures accelerate the development of conformable electronic skins (e-skins) and multimodal test manikins (RQ3). To ensure interlaboratory reproducibility within the current ISO 10218-2:2025 standard, the text defines imperative metrological parameters. These parameters strictly include frequency response, calibration protocols, and volumetric mapping of inertial masses (RQ4). Furthermore, the analysed publications were systematically stratified into distinct technological categories, strictly reflecting their primary engineering domains, ranging from empirical metrological evaluation and sensor hardware design to advanced numerical modeling. Finally, the vision for future research anticipates a definitive shift toward proactive anti-collision technologies, encompassing Artificial Intelligence (AI), machine vision, and Augmented Reality/Virtual Reality/Mixed reality (AR/VR/MR). Future methodologies must also consider demographic anisotropies and the cognitive fatigue of the human operator.

## 1. Introduction

The integration of collaborative robots (cobots) into industrial architecture determines the structural transformation of manufacturing processes. In the context of the transition toward the Industry 5.0 paradigm, the status of the human operator is redefined from a passive monitoring subject to an active and cooperative node within the production chain [1]. This anthropocentric approach accentuates the symbiosis of human cognition and adaptability with robotic precision. Consequently, it establishes collaborative robotics as a primary technological pillar of sustainable manufacturing systems [2]. The elimination of physical barriers creates an environment for direct Physical Human–Robot Interaction (pHRI). Within the Power and Force Limiting (PFL) regime, physical contact whether accidental or intentional is anticipated to inevitably occur [3].

Operator safety in these scenarios cannot be guaranteed solely by the passive reliability of the control system. Instead, it must be rigorously validated by quantifying contact events through the measurement of transmitted force and local pressure [4]. This validation requires specialized metrological instrumentation. Such devices must be capable of replicating the deformation characteristics of the human body with a high degree of biofidelity [5]. However, the accurate and reproducible objective measurement of these dynamic quantities faces multiple technological, methodological, and normative challenges [6].

Measurements of transient and high-speed contact events are typically heavily distorted by the nonlinear viscoelastic response of commercial test polymers. These synthetic materials do not adequately reflect the actual damping properties and the frequency-dependent elastic modulus of human soft tissues [7]. Therefore, achieving precise and reproducible objective measurements encounters a complex network of metrological barriers. The absence of strictly defined boundary conditions for the experimental setup and the limited frequency response of conventional surface sensors generate an unacceptable degree of uncertainty across the entire measurement chain [8].

To eliminate metrological ambiguities and address the evolving normative landscape, it is imperative to establish exact definitions for the terminology utilized throughout this study. The term biofidelity denotes the capacity of an artificial system to replicate the mechanical impedance of human biological tissues under dynamic impact loading. Physically, this requires matching the specific spring and covering stiffness parameters to the distinct body region values mandated in ISO 10218-2:2025 [9]. Instrumentation achieving this replication was historically referred to in early literature as a “measurement device” under the ISO/TS 15066 [10] specification. With the progression of safety standards, the specific classification PFMD was codified. Originating in the RIA TR R15.806-2018 [11] technical report, this term was subsequently formalized in ISO/PAS 5672:2023 [12] and ISO 10218-2:2025. A PFMD is strictly defined as a standardized testing apparatus equipped with specified compressive elements and sensors, constrained by exact normative geometric and structural requirements designed to measure peak force and peak pressure.

Consequently, throughout this paper, a strict terminological dichotomy is maintained. The term PFMD is exclusively reserved for standardized sensors that fully comply with formal normative descriptions and are recognized as official measurement devices within the cited literature. Conversely, the terms biofidelic device or biofidelic instrumentation denote general, custom-designed, or experimental prototypes. While these experimental systems achieve biofidelity by accurately simulating biological mechanical responses, they operate outside the official normative certification process and deviate from the rigid structural topology mandated for a formal PFMD classification.

### 1.1. Evolution of the Normative Framework and Validation Methodology

Requirements for safety assessment and biomechanical limits have undergone rapid normative evolution over the past decade. In 2011, the ISO 10218-1 [13] and ISO 10218-2 [14] standards defined the fundamental prerequisites for collaborative deployment. The publication of the ISO/TS 15066 technical specification in 2016 represented a primary milestone. This document formalized four specific collaboration methods. Furthermore, it established the first exact biomechanical threshold values for 29 distinct human body regions during quasi-static and transient contacts within the PFL regime [10].

The absence of a unified calibration and measurement methodology for the metrological instrumentation necessitated the creation of the RIA TR R15.806-2018 technical report for the North American market. This document specified evaluation procedures for biomechanical limits and defined explicit requirements for PFMD sensor placement [11]. These procedures were subsequently expanded and internationally harmonized within the ISO/PAS 5672:2023 standard [12].

**Figure 1:**
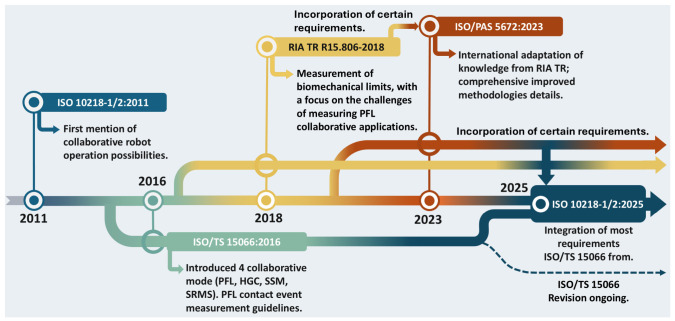
Timeline detailing the evolution of normative safety requirements governing Power and Force Limiting collaborative applications from 2011 to 2026.

This continuous evolution recently culminated in the publication of the updated ISO 10218-1:2025 and ISO 10218-2:2025 standards [9,15]. These revised standards fully integrate the primary safety premises of the original ISO/TS 15066 specification, alongside selected methodological procedures from the RIA and ISO/PAS documents. This integration establishes a unified, global framework for safety validation. Concurrently, the original ISO/TS 15066 undergoes a profound revision to reflect emerging technological realities. The complete chronological evolution of the normative framework since 2011 is illustrated in Figure 1.

The revised ISO 10218:2025 [9,15] standard introduces structural and technical modifications to reflect contemporary industrial operations, a transition thoroughly deconstructed in recent comparative analyses [16]. It explicitly addresses the domain of cybersecurity, mandating risk assessments for IT/OT vulnerabilities, implementing data checksums, and enforcing the deactivation of unused network ports. The terminology has been expanded to formally encompass mobile robotics, while simultaneously providing a strictly defined list of exclusions for which the standard is not applicable. A major structural modification is the transition of the hazard checklist from a normative requirement to an informative annex; the safety evaluation is now entirely dependent on a comprehensive risk assessment process strictly governed by the ISO 12100:2010 [17] methodology. Furthermore, the standard unifies the format of technical documentation by normatively requiring compliance with ISO 20607:2019 [18] for instruction handbooks. For the first time, it also acknowledges psychological factors, such as operator stress, as relevant residual risks. However, despite these modernizations, the standard remains highly conservative regarding safety verification. It strictly dictates physical measurements for validating biomechanical limits and explicitly prohibits the substitution of physical testing with simulations or mathematical estimations. While computational simulations are marginally acknowledged as a permissible tool for identifying potential collision points, the rigid insistence on physical validation underscores a persistent limitation in the normative framework, leaving extensive areas open for future research in virtual safety analytics. A comprehensive synthesis of these primary normative modifications is systematically detailed in Table 1.

### 1.2. Motivation for the Study and Research Questions

Despite a robust normative framework, both industrial and laboratory practices reveal significant data dispersion within the collision measurement process itself. Recent review studies primarily focus on algorithmic models or the extraction of physiological data regarding tissue damage [19,20,21]. However, the current literature lacks critical synthesis systematically addressing the physical limitations and characteristics of the measurement chain. The present study eliminates this metrological deficit. Concurrently, it expands the analytical scope to include a longitudinal evaluation of research trends. This integrated temporal analysis exactly quantifies the publication dynamics of the field and maps the historical correlation of the addressed problems. Furthermore, it identifies the ongoing technological shift from the initial verification of normative thresholds toward current challenges in advanced sensor instrumentation.

Therefore, the primary objective of this article is to provide a comprehensive systematic review utilizing the Web of Science and Scopus databases. This review is intended for both the research community and application engineers engaged in the safety validation of PFL applications. The text maps the trajectory from the early stages of collaborative robotics and the initial experimental efforts associated with the ISO/TS 15066 publication. It subsequently covers the development of non-commercial sensor prototypes and alternative methodologies, culminating in current sophisticated spatial safety analyses of robotic manipulators.

The formulation of the specific Research Questions (RQs) closely follows the longitudinal research trends and the exact metrological sequence dictated by the ISO/PAS 5672 and ISO 10218-2 standards. These normative documents inherently divide safety validation into discrete physical domains: the material limits and biofidelity of the instrumentation, the specific boundary conditions of the experimental setup (rigid versus unconstrained testing), the technological reliability of surface detectors, and the final exact standardization of the acquired data. Consequently, to systematically assess the state of the art, identify technological gaps, and quantify the degree of resolution across these specific procedural phases, the following Research Questions (RQs) were formulated:

- RQ1: How do the material architecture and structural design of current PFMD or biofidelic measurement devices limit their frequency response and escalate the overall measurement uncertainty during high-speed transient contacts?
- RQ2: What is the effect of experimental boundary conditions (absolutely rigid sensor fixation versus compliant and pendulum-based systems) on kinetic energy dissipation and the subsequent error in detecting maximum pressure spikes?
- RQ3: In which specific dynamic regimes do commercially available surface pressure sensors physically fail, and which alternative instrumentation or non-commercial prototypes demonstrate superior biofidelity?
- RQ4: Which technical and metrological parameters must be strictly standardized to achieve objective interlaboratory data reproducibility during safety validation under the former ISO/TS 15066 and the current ISO 10218-2:2025 standards?

### 1.3. Categorization of Research Domains

The volume of retrieved scientific literature reflects the multidisciplinary nature of the investigated problem. To systematically address the formulated research questions, the analysed publications were classified into five complementary categories. Each category represents a distinct engineering or scientific discipline.

This dual taxonomy—utilizing both specific RQs and thematic categories—establishes a highly structured orientation matrix for the reader. While the RQs serve to explicitly evaluate the state of the art and quantify how effectively a specific safety validation problem is physically resolved, the categorization functions as a direct spatial map of the multidisciplinary landscape. This separation enables immediate identification of a study’s orientation: the technical categories define exactly what methodological approach and engineering domain the publication utilizes (e.g., numerical modeling versus hardware design), thereby providing an exact overview of the specific tools applied to address the broader research questions.

- Category 1: Hardware design and development of unconventional sensor technology. These studies focus exclusively on the construction of physical measurement devices, artificial sensory skins, and anthropomorphic test manikins. The works critically analyse the implementation of piezoelectric films, optical fibres, and variable-stiffness mechanisms. The primary objective is to eliminate signal latency and acquire high-fidelity data through functional physical prototypes.
- Category 2: Metrological evaluation and normative methodology. This category encompasses research analysing systematic errors and uncertainties within measurement chains. It also evaluates the influence of experimental boundary conditions and the deficits of existing standardizations. Typical contributions include comparative analyses of certified sensors and the exact quantification of differences between rigid and compliant fixation. Furthermore, these studies investigate the physical influence of impactor geometry. The goal is to formulate more rigorous testing procedures to guarantee strict measurement reproducibility.
- Category 3: Numerical modeling and computational approximation. This research complements physical collision tests by utilizing the Finite Element Method (FEM), multi-body dynamics, machine learning algorithms, or spatial mapping techniques. These computational methods serve the exact prediction of transient force peaks. They also enable robust data extrapolation across diverse experimental setups.
- Category 4: Algorithmic and mechatronic mitigation of contact forces. These publications orient toward active or passive modifications of the robotic manipulator itself. Typical interventions include the integration of low-inertia actuators, the application of passive damping covers, or the design of energy-management control loops aimed at the physical reduction in transferred kinetic energy.
- Category 5: Biomechanical characterization of tissues and human perception. This domain covers the physical and medical analysis of the deformation behaviour of human tissue and the determination of pain thresholds. These studies establish foundational data for determining nonlinear viscoelastic parameters. Consequently, they expose the physical limitations of simplified normative models and commercial test polymers during high-speed transient impacts.

The comprehensive evaluation within this article also incorporates visualization through cross-correlation matrices. These matrices graphically represent the intersection between the defined research questions and the investigated categories. Simultaneously, they document the longitudinal evolution of the scientific community’s focus regarding specific aspects of pHRI metrology.

## 2. Methodology

Two primary citation databases, Web of Science (WoS) and Scopus, were selected for the identification of relevant literature. The search query utilized Boolean operators to comprehensively cover three fundamental technical pillars. These pillars comprise (1) the domain of collaborative robotics, (2) the specification of contact events within the normative framework, and (3) metrological instrumentation.

### 2.1. Search Strategy and Dataset Identification

The term “biofidelic” was deliberately integrated into the search string. This term served as the primary descriptor for measurement devices in the literature published prior to the establishment of the standardized acronym PFMD (Power and Force Measuring Device). The temporal scope of the search was restricted to the period between 2011 and 2026. This timeframe corresponds to the publication of the ISO 10218-2:2011 standard, which initially formalized the operational parameters for collaborative robotics [14].

**Search string for the Web of Science database (Advanced Search):** TS = ((“collaborative robot*” OR “cobot*” OR “human-robot interaction” OR “HRI” OR “HRC” OR “pHRI” OR “human-robot collaboration” OR “collaborative application*” OR “industrial robot*” OR “industrial manipulator*”) AND (“power and force limiting” OR “PFL” OR “collision*” OR “impact force*” OR “impact test*” OR “physical contact” OR “contact event*” OR “clamping” OR “pinching” OR “ISO 15066” OR “ISO TS 15066” OR “biomechanical threshold*” OR “robot safety” OR “robotic safety” OR “mechanical hazard*” OR “safety validation”) AND (“sensor*” OR “measure*” OR “biofidelic*” OR “biofidel*” OR “compliance” OR “damping” OR “validate*” OR “test method*” OR “experimental test*” OR “experimental study” OR “protective skin” OR “PFMD”)).

**Search string for the Scopus database (Advanced Search):** TITLE-ABS-KEY(“collaborative robot*” OR “cobot*” OR “human-robot interaction” OR “HRI” OR “HRC” OR “pHRI” OR “human-robot collaboration” OR “collaborative application*” OR “industrial robot*” OR “industrial manipulator*”) AND TITLE-ABS-KEY(“power and force limiting” OR “PFL” OR “collision*” OR “impact force*” OR “impact test*” OR “physical contact” OR “contact event*” OR “clamping” OR “pinching” OR “ISO 15066” OR “ISO/TS 15066” OR “biomechanical threshold*” OR “robot safety” OR “robotic safety” OR “mechanical hazard*” OR “safety validation”) AND TITLE-ABS-KEY(“sensor*” OR “measure*” OR “biofidelic*” OR “biofidel*” OR “compliance” OR “damping” OR “validate*” OR “test method*” OR “experimental test*” OR “experimental study” OR “protective skin” OR “PFMD”).

The initial query yielded 1252 records in WoS and 2474 records in Scopus. The subsequent application of inclusion filters—specifically document type, English language, and the defined temporal window—reduced the datasets to 1161 and 2061 records for WoS and Scopus, respectively. Regarding database categorization, no restrictive subject filters were applied. This methodological decision prevented the exclusion of relevant multidisciplinary studies intersecting robotics, metrology, and biomechanics.

### 2.2. Study Selection Process

Following the consolidation of the datasets, both automated and manual deduplication processes were applied based on DOIs, article titles, and author names. This procedure removed a total of 2123 duplicate records. Consequently, 1099 unique publications proceeded to the initial screening phase.

Two independent experts conducted the evaluation. Any discrepancies during the evaluation process were resolved through iterative expert discussion until full consensus was reached. Consequently, the involvement of a third independent arbitrator was not required during the selection process.

- Title and Abstract Screening: The inclusion criteria were stratified based on the technological evolution of the field. An absolute prerequisite for inclusion was a demonstrable correlation with Physical Human–Robot Interaction (pHRI) or a direct application within collaborative scenarios utilizing the Power and Force Limiting (PFL) regime. For literature published prior to 2016, the evaluation focused on the presence of experimental measurement data or the engineering design of sensors for the local detection of contact pressures and forces. A direct comparison with exact biomechanical limits was not strictly required for these older studies. Conversely, publications issued after 2016 required explicit adherence to formalized normative values. These recent studies had to demonstrate rigorous evaluation methods for transient and quasi-static phenomena, precise identification of measurement points, or the advanced development and validation of instrumentation for the objective mapping of collision forces and pressures. Following this phase, 101 publications proceeded to the next stage.
- Full-Text Screening: For final inclusion, the article had to demonstrably present validated experimental measurements or an exact sensor design supported by empirical data. Alternatively, it required an objective comparison with the threshold values defined in ISO/TS 15066:2016 and ISO 10218-2:2025, or the direct measurement of contact forces during pHRI. This phase rigorously verified the premises indicated within the titles and abstracts. Therefore, it effectively eliminated false-positive matches and potential methodological misinterpretations. Based on these strict parameters, 68 full texts were ultimately selected for data extraction and subsequent technical synthesis. A comprehensive tabular synthesis, explicitly detailing the specific methodological and metrological parameters of each included publication, is provided in Appendix A.

**Figure 2:**
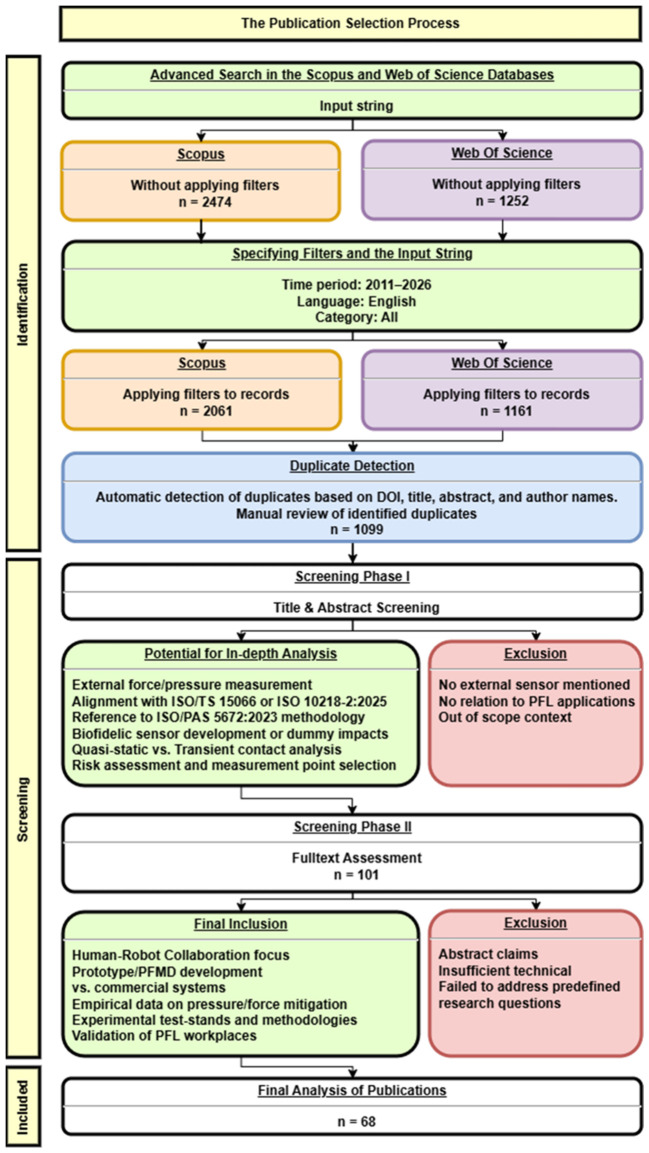
PRISMA diagram detailing the systematic literature identification and screening process.

The entire selection process, including the explicit rationale for excluding publications during the full-text screening phase, is schematically illustrated in the PRISMA diagram (see Figure 2).

### 2.3. Data Normalization and Classification

The analysed literature exhibited severe terminological inconsistency. Authors frequently demonstrated inconsistent usage of related terms, such as HRI, HRC, and pHRI, alongside divergent pluralization conventions. To mitigate this statistical discrepancy, a computational semantic normalization process was executed prior to the generation of correlation matrices and network diagrams.

A dedicated Python 3.14.0 script was developed to perform linguistic lemmatization and the standardization of acronyms (e.g., merging the expanded phrase “power and force limitation” with the exact acronym “PFL”). Subsequently, the normalized terms were algorithmically clustered into six distinct technical domains with assigned classifications:

- Safety & Standards (Normative framework and risk assessment);
- Interaction & Collision (Contact mechanics and interaction typologies);
- Hardware & Robots (Mechatronic architectures and physical platforms);
- Sensing & Measurement (Metrological instrumentation);
- Modeling & Biomechanics (Computational approximations and tissue behaviour);
- Other/Specific (Miscellaneous technical domains not classifiable into the aforementioned primary categories).

### 2.4. Quality Assessment and Evidence Weighting Framework

Conventional medical “Risk of Bias” assessment tools are structurally inapplicable to mechatronic and metrological engineering research, as procedures typical for the medical domain, such as double-blinding or randomized controlled trials, are usually impossible to implement in these technical fields. Furthermore, systematic research by Kitchenham et al. [22,23] has explicitly confirmed that quality checklists derived from the medical domain are inappropriate for technology-intensive engineering studies. Therefore, following the established guidelines for systematic literature reviews in engineering disciplines proposed by Kitchenham et al. [22,23], which advocate for developing tailored quality instruments that specifically consider the empirical study type and its scope, a domain-specific Quality Assessment (QA) table was developed. This framework ensures that the formulated technological trajectories and normative recommendations are derived strictly from highly validated, contemporary scientific evidence, preventing the analytical equivalence of preliminary historical conference papers with recent high impact journal publications.

Each of the 68 included publications was quantitatively evaluated using a Total Quality Score (TQS), ranging from 3 to 9 points. The TQS aggregates three specific evaluative pillars:

- Source Rigor (SR): Evaluates the strictness of the peer-review process. Articles published in Q1/Q2 impact-factor journals are assigned the maximum score (3 points). Publications in Q3/Q4 journals receive 2 points, while conference proceedings receive 1 point. This parameter objectively isolates scientific rigor without introducing citation time-window bias against recently published state-of-the-art papers.
- Methodological Rigor (MR): Evaluates experimental validity and metrological purity. Maximum weight (3 points) is awarded to physical experiments featuring fully defined boundary conditions (e.g., kinematic compliance) and rigorous statistical data evaluation. Validated numerical simulations (FEM) or physical tests with limited statistical analysis receive 2 points. Purely virtual simulations lacking physical benchmarking or preliminary Proof-of-Concept prototypes receive 1 point. For secondary studies (review articles), Methodological Rigor is evaluated based on systematic reproducibility. Maximum weight (3 points) is assigned to Systematic Literature Reviews (SLRs) adhering to strict methodological protocols (e.g., PRISMA), featuring explicit search strategies, inclusion/exclusion criteria, and formal quality assessment of primary studies. Comprehensive state-of-the-art reviews with defined research questions and thematic synthesis, but lacking a fully replicable search protocol, receive 2 points. Narrative or informal literature reviews without defined methodology receive 1 point.
- Temporal Relevance Index (TRI): Given the rapid evolution of sensory hardware, a Temporal Relevance Index was implemented to filter obsolete metrological deficits. Publications from 2023–2026, reflecting contemporary hardware paradigms and the transition toward ISO 10218-2:2025, receive 3 points. The 2017–2022 period, reflecting the technological absorption of ISO/TS 15066, receives 2 points. Historical studies (2011–2016) documenting obsolete hardware configurations receive 1 point.

The quality assessment was performed independently by two researchers, applying the exact consensus protocol previously defined in Section 2.2.

Determined by the calculated mathematical sum (TQS = SR + MR + TRI), the publications were stratified into three evidence levels: High Confidence (8–9 points), Moderate Confidence (5–7 points), and Low/Historical Relevance (3–4 points). During the technical synthesis (Section 4 and Section 5), outdated hardware limits derived from Low Relevance papers are treated strictly as historical context, whereas the formulation of future proactive safety trajectories and normative standardizations relies exclusively on High Confidence literature. The resulting evidence levels for all processed studies are explicitly documented in Appendix B.

## 3. Results

Prior to conducting the in-depth technical synthesis and addressing the formulated research questions, a bibliometric analysis and descriptive statistics were executed on the extracted dataset. The primary objective of this section is to quantify the scientific community’s engagement with the subject matter. Furthermore, it visualizes the correlation linkages between individual technological domains.

### 3.1. Bibliometric Analysis of the Retrieved Sample

**Figure 3:**
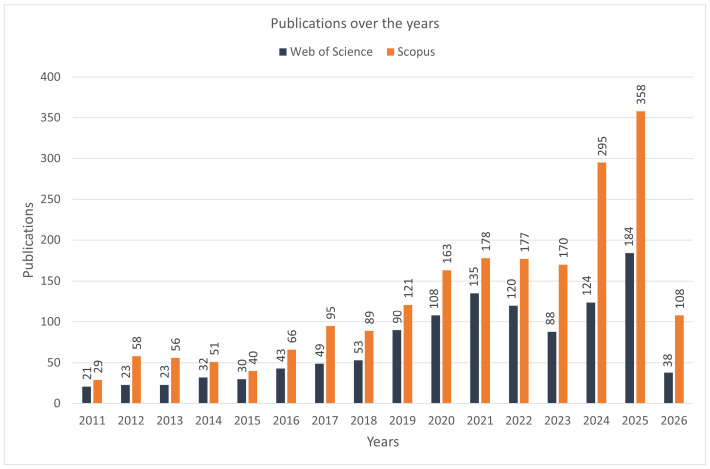
Annual distribution of publications indexed within the Web of Science and Scopus databases spanning the 2011–2026 period.

The quantitative analysis of publication activity demonstrates a stable, long-term upward trend. The volume of literature regarding safety and measurement in collaborative robotics expands annually. The only slight correction and subsequent decline in publication dynamics occurred between the years 2022 and 2023 (see Figure 3).

Regarding document typology, original scientific articles dominate both databases. Conference proceedings closely follow this primary category. Review articles and Early Access publications constitute a marginal fraction of the WoS dataset. Conversely, conference reviews and traditional review papers supplement the statistical distribution within Scopus.

**Figure 4:**
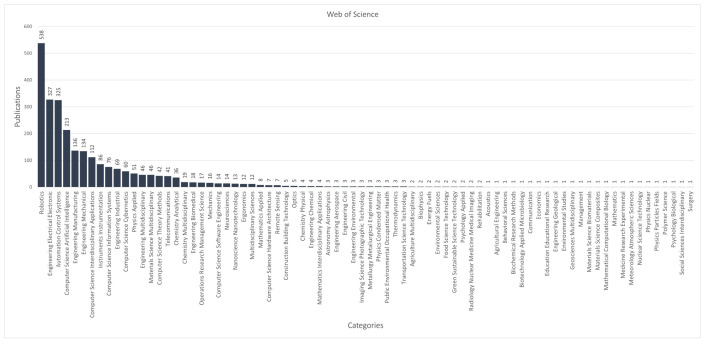
Distribution of the extracted publications across specific Web of Science research categories.

**Figure 5:**
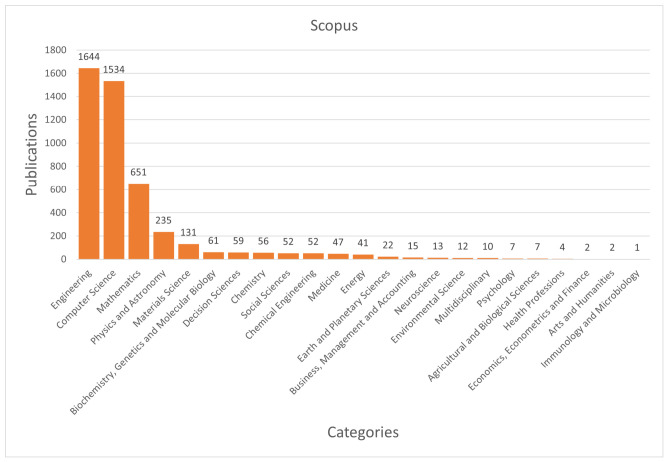
Distribution of the extracted publications across specific Scopus research categories.

The distribution of publications across subject categories confirms the strictly technical and applied orientation of the investigated topic. Within the WoS database, the “Robotics” category exhibits the highest representation. The categories “Engineering Electrical Electronic” and “Automation Control Systems” follow with a significant statistical distance. The Scopus database demonstrates peak saturation within the broader “Engineering” category. The “Computer Science” discipline follows with a marginal gap (see Figure 4 and Figure 5).

To identify the primary semantic nodes across the retrieved records, a network graph mapping keywords and topics was constructed. Only terms with an occurrence frequency of six or higher were retained. Lower frequency thresholds rendered the graphical topology unreadable.

**Figure 6:**
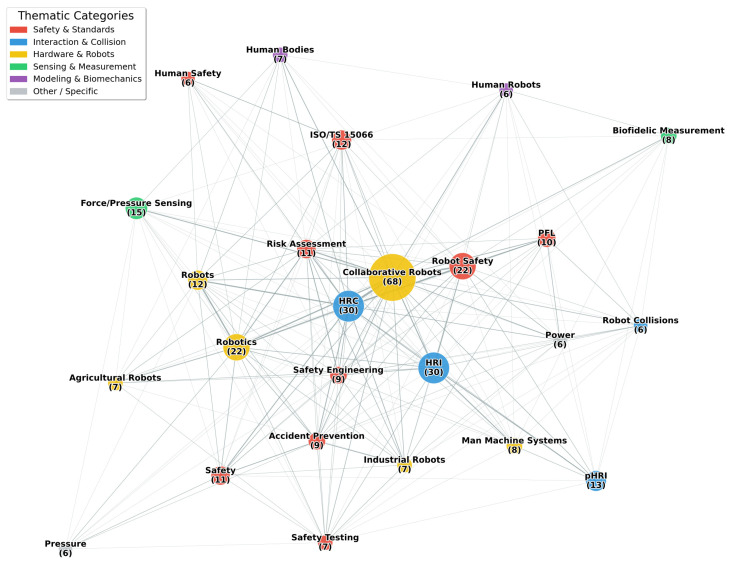
Network diagram visualizing the thematic interconnectivity among the selected publications.

**Figure 7:**
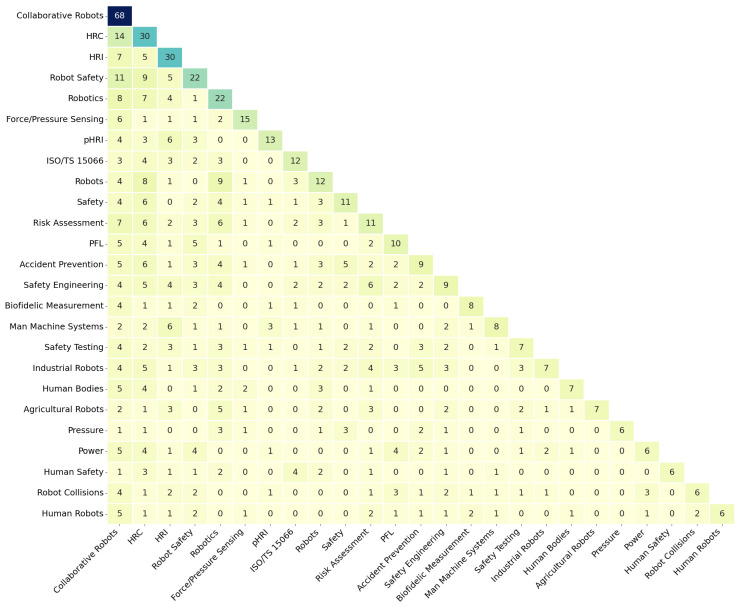
Correlation matrix quantifying the thematic intersections and interconnectivity within the selected publications.

The terms “Collaborative Robots”, “HRC”, “HRI”, “Robot Safety”, and “Robotics” exhibit the highest semantic centrality. A correlation matrix complements this network topology. This matrix mathematically quantifies the degree of co-occurrence among specific terms across the publications, thereby providing an exact overview of topical intersections (see Figure 6 and Figure 7).

### 3.2. Descriptive Statistics of the Final Sample

**Figure 8:**
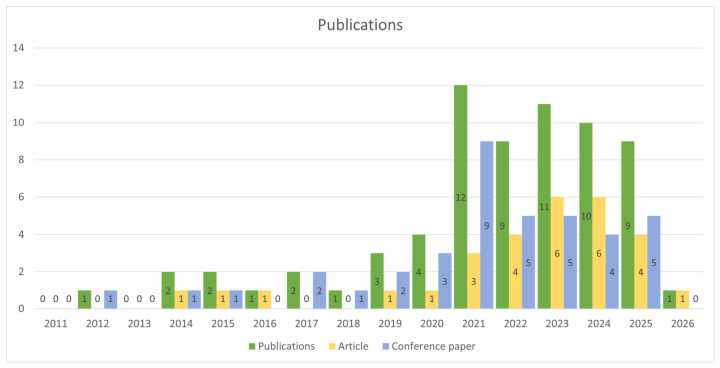
Longitudinal progression of the selected publications (2011–2026), specifically detailing the distribution between journal articles and conference proceedings.

While the broad bibliographic sample demonstrates continuous growth, the rigorously filtered final selection of 68 publications exhibits a divergent temporal dynamic. These publications successfully passed the two-stage screening process by presenting empirical measurements, PFMD architectures, and normative validation, revealing a sharp quantitative peak in experimental studies in 2021. This peak reflects the latency necessary for developing new experimental methodologies and executing comprehensive data collection following the 2016 publication of the ISO/TS 15066 technical specification. Currently, the publication frequency of these strict metrological studies displays a slight downward trajectory. This decline indicates a gradual technological saturation of the initial exploratory phase of pHRI research (see Figure 8).

Within the rigorously filtered final dataset, the distribution shifts, with international conference proceedings constituting the primary format (39 records). Peer-reviewed articles published in impact-factor journals supplemented this primary set (29 records).

**Figure 9:**
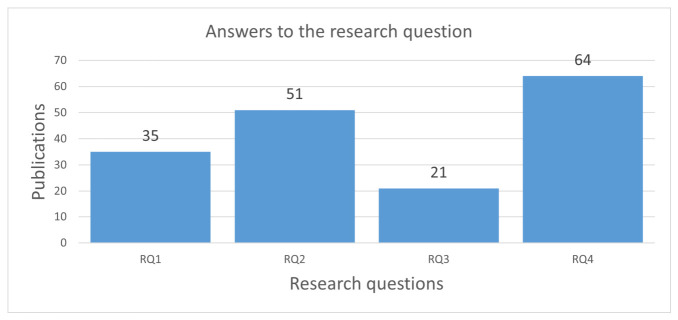
Quantitative distribution of the selected publications categorized by the defined Research Questions (RQs).

Saturation of Research Questions and Technical CategoriesAn in-depth analysis of the final dataset revealed significant structural asymmetries in the focus of contemporary research. Mapping the extracted articles against the formulated Research Questions (RQ1–RQ4) demonstrated that the large majority of publications target RQ4 (Standardization and metrological parameters for validation). Conversely, RQ3 exhibits the most substantial research gap. This specific question investigates the physical failure of commercial surface sensors and the subsequent development of non-commercial, high-biofidelity prototypes (see Figure 9).

**Figure 10:**
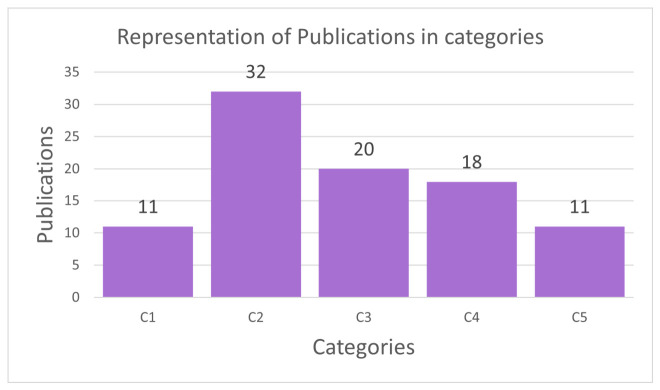
Quantitative distribution of the selected publications classified by specific technological categories.

The quantitative distribution across the five defined technical categories (C1–C5) corroborates this trend. Category 2 (Metrological evaluation and normative methodology) commands the most robust publication foundation. In contrast, Category 1 (Hardware design and sensor development) and Category 5 (Biomechanical characterization) exhibit the lowest degree of publication saturation (see Figure 10).

Correlation Analysis of the Investigated Domains

**Figure 11:**
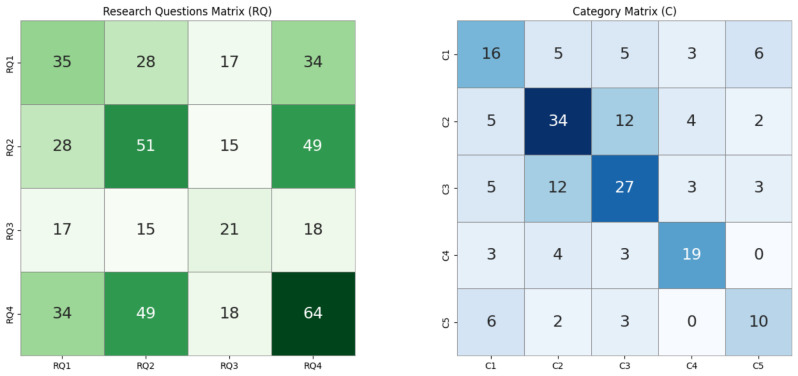
Cross-reference matrix correlating the defined Research Questions (RQs) with the established technological categories.

To expose the internal linkages within the final dataset, correlation matrices were constructed. These matrices map the topical intersections across the formulated Research Questions and technical categories within individual publications (see Figure 11). Among the Research Questions, the strongest correlation emerged between RQ2 (Influence of experimental setup and fixation) and RQ4 (Standardization). This robust linkage logically implies that metrological standardization efforts fundamentally depend on the exact definition of experimental boundary conditions. Consequently, the pursuit of interlaboratory data reproducibility lacks a valid physical foundation if the normative framework fails to strictly define the kinematic compliance and the kinetic energy dissipation mechanisms of the entire test chain. Ensuring objective certification compliance imperatively demands the comprehensive unification of both rigid and unconstrained transient impact scenarios. This metrological necessity forces researchers to address both paradigms simultaneously.

Conversely, the weakest correlation manifested between RQ3 (Deficits of commercial sensor technology and prototype development) and RQ2 (Influence of experimental setup). This analytical divergence stems from the fundamentally different methodological foci of these two domains. Research teams develop innovative metrological hardware with superior biofidelity primarily orient their efforts toward the internal material architecture, piezoresistive properties, and electronic frequency response of the novel sensor. To rigorously isolate these internal variables, empirical studies typically verify their prototypes under strictly simplified and static boundary conditions. Consequently, these researchers completely abstract their validation from the parallel analysis of the macroscopic kinematics characterizing the test stands. This finding reveals a significant paradigm gap: the development of novel sensory hardware and the research regarding macroscopic collision dynamics currently operate as two parallel, mutually unintegrated engineering disciplines.

Although topical intersections across the five defined categories were less frequent than among the Research Questions, the correlation matrix revealed highly relevant synergistic nodes. The highest co-occurrence was identified between Category 2 (Metrological evaluation) and Category 3 (Numerical modeling). This strong intersection is fully justified from both a physical and methodological perspective. Given the high economic demands, time constraints, and spatial limitations inherent to physical impact tests (C2), research teams endeavour to extrapolate measured discrete data utilizing surrogate computational models and the Finite Element Method (C3). However, any developed numerical model strictly necessitates empirical metrological validation. Therefore, these two domains constitute an inseparable evaluation chain within contemporary research.

The second most prominent intersection involves the joint occurrence of Category 1 (Hardware design) and Category 5 (Biomechanical characterization). This phenomenon closely reflects the engineering reality of biofidelic sensor development. Authors constructing novel, non-commercial hardware for precise measurement (C1) are inherently compelled to integrate empirical data regarding the nonlinear viscoelastic response of human tissues (C5). This data integration is strictly required to demonstrably and quantitatively validate the biofidelity of their mechatronic architectures against a biological benchmark.

At the opposite pole of the correlation spectrum, a zero-occurrence overlap was identified between Category 4 (Algorithmic and mechatronic mitigation) and Category 5 (Biomechanical characterization). This absence of coexistence indicates a strict methodological barrier. Studies oriented toward the active reduction in transferred kinetic energy on the manipulator side do not currently incorporate direct experimental tissue characterization. Instead, they process biomechanical limits as fixed, static constants derived directly from normative documents. This identified metrological deficit exposes a substantial scope for future research, which should integrate the dynamic viscoelastic response of human tissues directly into adaptive, proactive anti-collision algorithms.

Longitudinal Distribution of Research Questions and Technological Domains

**Figure 12:**
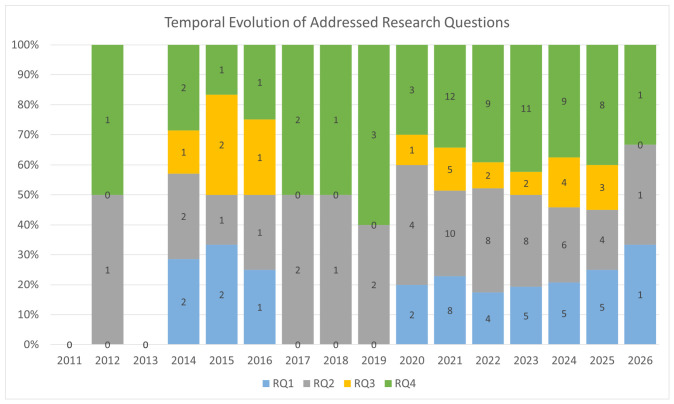
Longitudinal distribution (2011–2026) of the selected publications, stratified by their corresponding Research Questions (RQs).

**Figure 13:**
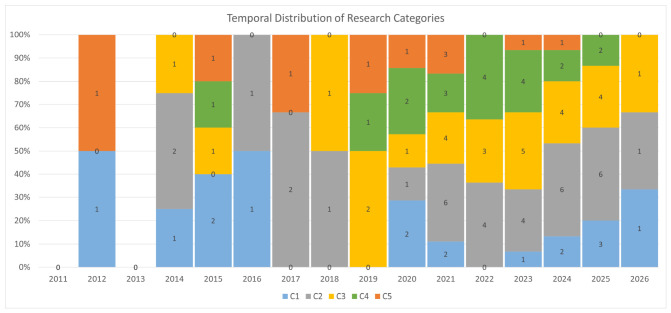
Longitudinal distribution (2011–2026) of the selected publications, stratified by their designated technological categories.

An analysis of the temporal evolution within the final sample of 68 publications (see Figure 12 and Figure 13) provides exact insight into the shifting research priorities within the domain of pHRI metrology. Regarding the formulated Research Questions, the longitudinal distribution exhibits a steep publication peak in 2021 across all monitored parameters. This phenomenon represents a distinct technological latency. It reflects the five-year cycle required for the design, execution, and publication of complex, validated experiments following the 2016 release of the ISO/TS 15066 technical specification.

The extracted data demonstrates the persistent dominance of RQ4 (Standardization and reproducibility). This specific inquiry culminated in 12 publications in 2021 and maintains a high degree of saturation in contemporary research. Simultaneously, RQ2 (Influence of experimental setup) exhibits a robust historical trend. This trajectory proves the growing awareness within the scientific community that kinetic energy dissipation within test stands fundamentally compromises measurement validity. Conversely, RQ3 (Deficits of commercial sensor technology) remains longitudinally underrepresented, reaching a maximum of merely five publications in 2021. This metrological deficit confirms a persistent reliance on commercial “black-box” solutions, conspicuously lacking profound hardware critique.

This identified trend fully corresponds to the publication distribution across the technical categories (C1–C5). Category 2 (Metrological evaluation) represents the most historically saturated domain, maintaining stable publication activity with six records annually during 2021, 2024, and 2025. This consistency proves that the engineering field continuously struggles with the practical interpretation and application of strict normative requirements. However, Category 3 (Numerical modeling) demonstrates a notable longitudinal shift. Prior to 2020, the utilization of surrogate models and the Finite Element Method (FEM) remained highly sporadic. Subsequently, this category recorded a significant surge between 2022 and 2024, peaking at five publications in 2023. This paradigm shift indicates a systematic effort by research teams to reduce the economic and temporal burdens of physical impact tests through advanced computational approximations.

Category 5 (Biomechanical characterization) occupies the opposite end of the distribution spectrum. This domain recorded a moderate increase proportional to the initial implementation of the safety standards, peaking in 2021. Nevertheless, its occurrence has declined to a notable minimum in recent years (2024–2026). This decline signals that the fundamental physiological pain thresholds and tissue tolerances are currently considered sufficiently mapped. Consequently, the research focus has definitively transitioned from biological mapping toward the optimization of metrological hardware and computational models. Throughout the entire monitored period, Category 1 (Hardware design and sensor development) maintains a marginal yet stable representation of one to three publications per year. This constant sparsity directly reflects the severe technological and financial barriers inherently associated with developing functional physical prototypes of biofidelic metrological instrumentation.

### 3.3. Evidence Level Distribution

Following the application of the defined Total Quality Score (TQS) methodology, the final dataset of 68 publications was quantitatively stratified to determine its overall evidence weight. The evaluation revealed that 21 publications achieved the High Confidence level (TQS 8–9). This subset primarily consists of recent, extensively validated empirical studies published within high-impact journals (2023–2026), thus forming the most robust foundation for formulating future research trajectories.

A total of 42 publications were classified as Moderate Confidence (TQS 5–7), predominantly representing advanced conference proceedings or numerical simulations lacking complete physical benchmarking. The remaining 5 publications fell into the Low/Historical Relevance tier (TQS 3–4). These records typically encompass preliminary conceptual designs or older empirical studies (2011–2016). While metrologically inadequate for current normative standards, this specific subset remains analytically highly valuable for mapping the historical progression of hardware defects and documenting the evolution of measurement uncertainty within the pHRI domain. The exact TQS classification for each specific publication is explicitly detailed in Appendix B.

## 4. Results Technical Synthesis and Metrological Analysis

The subsequent chapter constitutes the analytical core of this study, providing a systematic deconstruction of the extracted datasets. Its structural organization follows the structure of the four defined Research Questions (RQ1–RQ4). This framework logically guides the reader through the complete procedural chain necessary for the objective safety evaluation of collaborative applications operating within the Power and Force Limiting (PFL) regime.

Section 4.1 analyses the internal hardware limitations and material architecture of contemporary PFMD and biofidelic instrumentation. Specifically, it investigates their unintended function as nonlinear mechanical filters during high-speed transient impacts.

Section 4.2 shifts the research focus toward the macroscopic level to quantify the physical influence of experimental boundary conditions. Primarily, it evaluates the differential impact of rigid versus compliant kinematic fixation on total kinetic energy dissipation and signal amplitude distortion.

Section 4.3 subsequently subjects conventional commercial sensor technology (PFMD) to a rigorous metrological critique. Furthermore, it introduces innovative laboratory prototypes that demonstrate a substantially higher degree of biofidelity.

Finally, Section 4.4 aggregates the identified metrological deficits into a comprehensive set of technical imperatives. The strict standardization of these parameters represents a key requirement for achieving objective interlaboratory data reproducibility within the context of the current ISO 10218-2:2025 standard.

To ensure the objective formulation of metrological limitations, the following synthesis integrates the evidence weighing established in Section 2.3. Highly validated structural imperatives and fundamental physical deficits are derived exclusively from High Confidence literature, whereas theoretical approximations and simulation-based prototypes (Moderate Confidence) are treated strictly as trajectories requiring further empirical verification.

### 4.1. Material Architecture and the Dynamic Limits of PFMDs and Biofidelic Instrumentation

The material architecture and mechatronic design of contemporary standardized sensors fully complying with formal normative descriptions such as ISO/PAS 5672:2023 and ISO 10218-2:2025—actively function as complex nonlinear filters, structurally dictating a massive fraction of metrological uncertainty during the evaluation of high-speed transient impacts. Empirical data from Samarathunga et al. [21,24] (High Confidence) rigorously confirms that when an impact duration falls below the internal time constant of a standard PFMD, the device mathematically forces a systematic underestimation of force peaks. To explicitly quantify this, they model the sensor dynamics as $f_{i}=m\ddot{x}\left(t\right)+c\dot{x}\left(t\right)+kx(t)$, which yields a second-order transfer function:

(1) $$\frac{F_{o}}{F_{i}}=\frac{1}{\frac{s^{2}}{\omega_{n}^{2}}+\frac{2\xi }{\omega_{n}}s+1}$$

They provide undeniable physical evidence that the inertial mass *m* of the moving impact plate and internal linear guides restricts the overall frequency bandwidth, defined analytically as $f_{BW}=f_{n}\sqrt{\left(1-2\xi^{2}\right)+{\left(2\xi^{2}-1\right)}^{2}+1}$. With an experimentally validated damping ratio of only ξ≈0.07, the certified instrument transforms into a highly underdamped, oscillatory mechanical system. Furthermore, applying a Generalized Maxwell Model to the viscoelastic covering dictates a transfer function between contact deformation W and output force $F_{o}$ defined as $\frac{F_{o}}{W}=\;k\frac{B}{A\;+\;B}$, mathematically proving that an incorrect rheological architecture degrades the accurate tracking of rapid transient phenomena. Consequently, for impacts shorter than the sensor’s time constant τ ≈ 0.01 s, the physical mechanical filtration massively escalates the measurement uncertainty within any rigorous GUM framework. Conversely, while Zhu et al. [25] (Moderate Confidence) identified additional structural anomalies, such as idle strokes induced by the absence of rigid fixation and parasitic friction within guide bushings acting as unquantified kinetic energy dissipators in custom test setups, their findings lack rigorous mathematical quantification regarding the actual frequency response. Further empirical verification by Staab et al. [26] (Moderate Confidence) observed mechanical bounce-back within the linear mechanisms, yet their analysis primarily indicates an emerging testing trend requiring further physical validation rather than defining an absolute metrological limit.

This hardware inconsistency generates massive data dispersion. Scibilia et al. [27] and Zimmermann et al. [7] (High Confidence) provided highly rigorous interlaboratory data demonstrating that under identical impacts by a KUKA LBR iiwa robot, certified PFMDs output diametrically disparate forces with extreme variances. Specifically, these studies mathematically proved that amplitude distortion escalates primarily during high-speed unconstrained transient impacts due to differing dynamic spring stiffnesses and variable viscoelastic damping inherent to these normative architectures. While Zimmermann et al. [28] (Moderate Confidence) documented similar error rates, their analysis remains limited in its full metrological isolation of variables. Furthermore, D’Antona et al. [29] (High Confidence) explicitly quantified the physical saturation limit of commonly utilized tempered steel springs (C67S), demonstrating that a rigid topology mandated for formal PFMD classification fails (effective stiffness capped at $60\;\frac{N}{mm}$) when attempting to approximate more rigid human anatomical regions. From a signal conditioning perspective, they define the exact transfer functions governing the analog front-end, where the transimpedance amplifier output $V_{\mathrm{out}}\;=\;-V_{dd}\cdot \frac{R^{2}}{R^{1}}$ dictates that an incorrectly scaled resistor $R_{2}$ will physically clip transient peaks, inducing severe systematic errors. To mitigate electromagnetic interference common in industrial environments, they implemented a differential topology $V_{\mathrm{out}}\;=\;2V_{\mathrm{in}+}\;-\;V_{\mathrm{in}-}$ strictly required to eliminate common-mode noise from the expanded measurement uncertainty. The metrological unreliability extends beyond the sensor itself; research by Fischer et al. [30] and Schneider et al. [31,32] (Moderate Confidence) indicates that the macro-architecture of the test stand—specifically supplementary damping materials and unconstrained contact geometries—modulates uncertainty, although their conclusions currently lack absolute physical standardisation. Similarly, Schlotzhauer et al. [33] and Hüsing et al. [34] (Moderate Confidence) proposed modular impactors to control these boundary conditions, representing a preliminary testing method that strictly demands further empirical validation to definitively isolate complex structural defects.

The evolution of metrological limits is distinctly visible when contrasting contemporary standardized hardware with advanced, non-certified biofidelic instrumentation. Theoretical models, such as the NIR index evaluated by Cordero et al. [35] (Moderate Confidence), assumed an ideal sensor response without evaluating specific frequency bandwidths; while methodologically relevant for early modeling, they lack contemporary empirical validation and inherent biofidelity. Modern tissue simulation demands superseding these linear normative assumptions. Empirical research by Rajaei et al. [36] and Dagalakis et al. [37] (Moderate Confidence) attempted to define the hyper-viscoelastic deformation response of actual human tissue, establishing preliminary nonlinear kinetic energy dissipation parameters for pre-test calibration of advanced biofidelic devices. Conversely, Caneschi et al. [38] (High Confidence) rigorously demonstrated the exact limitations of current prototype design algorithms by defining the directional effective mass dynamically:

(2) $$m_{robot}\left(q,\hat{n}\right)=\frac{1}{{\hat{n}}^{T}\;J(q)M{\left(q\right)}^{-1}{J(q)}^{T}\;\hat{n}}$$

By applying the nonlinear viscoelastic Hunt–Crossley model $F(\delta ,\dot{\delta )}=k_{HC}\delta^{n}\left[1+\frac{3(1-c_{r})}{2}\frac{\dot{\delta }}{\left|{\dot{\delta }}_{0}\right|}\right]$, their optimization revealed that for transient impacts, the damping coefficient is mathematically negligible (restitution coefficient $c_{r}>\;0.94$), reducing the interaction to a purely elastic Hertzian model $F\left(\delta \right)=k_{H}\delta^{n}$. Consequently, their custom system generates a nearly perfect elastic impact that definitively fails to capture physiological reality. To correct systematic errors derived from fixed testing setups compliant with ISO/PAS 5672:2023, they mandate an uncertainty scaling factor $F_{eq}=F_{meas}\cdot \sqrt{\frac{m_{body}}{m_{body}+m_{robot}}}$. Furthermore, their FFT analysis localizes the dominant transient spectral power between 2 Hz and 68 Hz, isolating transient undershoot as a pure piezoelectric high-pass measurement artifact that must be addressed in precise GUM evaluations. The severe strain-rate dependency of silicone and urethane gels was identified by Hirata et al. [39] and Iki et al. [40] (Moderate Confidence), suggesting that outer covering layers of experimental biofidelic instrumentation actively function as nonlinear phase and amplitude modulators, albeit awaiting broader physical verification. Moreover, the current 1D normative approach exhibits strict physical insufficiency. Theoretical critiques by Valori et al. [20] (Moderate Confidence) regarding the inability of flat surface PFMDs to capture spatial energy redistribution are now robustly substantiated by empirical evidence; structural analyses by Fischer et al. [41] and Liu et al. [42] (High Confidence) successfully deconstruct this architectural deficit. Fischer et al. [41] expand the simplistic ISO model to incorporate exact functional dependencies for transient force $F_{t}=f(m_{H},\;v_{rel},m_{R}\left(q,u\right),k_{S},k_{D})$, proving that the damping stiffness $k_{D}$ and impactor geometry severely alter dynamics. They quantify a standard uncertainty of ±15 N for strain gauges and up to ±10% for pressure foils affected by structural bulging artifacts. Specifically, Liu et al. [42] integrated materials into a novel biofidelic device by replacing constant stiffness with a nonlinear geometric model $k_{i}=\gamma_{i}x^{2}$, which derives an exact transient force equation:

(3) $$F\left(t\right)=\gamma_{1}{\left(vt\right)}^{3}+\sum_{i=2}^{3}c_{i}v\left[1-exp\left(-\frac{\gamma_{i}}{c_{i}}v^{2}t^{3}\right)\right]$$

This mathematical formulation proves that viscous elements $c_{3}$ physically induce structural overshoot and ringing; the exponential dependence on $v^{2}$ and $t^{3}$ limits the frequency response during high-speed transients, causing GUM parameters to severely overestimate initial tissue stiffness.

Beyond material compliance, capturing the steep gradients of transient peaks imposes strict data acquisition constraints. Švarný et al. [43] (Moderate Confidence) defined a minimal required sampling frequency of 1000 Hz, a parameter definitively surpassed by the robust hardware architecture of D’Antona et al. [29] (High Confidence), who implemented FPGA technology within their custom biofidelic instrumentation. This deterministic parallel data acquisition rigorously mitigates aliasing, radically reducing Type A uncertainty in the time domain to achieve exact sampling rates between 10 kHz and 20 kHz. Conversely, conventional surface sensory foils, documented by Matthias et al. [44] (Low/Historical Relevance), exhibit nonlinear deformations and completely lack the capacity for absolute dynamic calibration required for certified PFMDs. Attempts to integrate pneumatic decoupling elements into experimental biofidelic devices, evaluated by Kim et al. [45] (Low/Historical Relevance), represent an obsolete approach that induces severe pneumatic latency, empirically verified to permanently destroy signal integrity by massively flattening force peaks by 32% to 52%. As industry transitions toward proprioceptive robotic anti-collision systems, studies by Kirschner et al. [46] and Rustler et al. [47] (Moderate Confidence) highlight that reaction latency remains subject to severe spatial anisotropy within Contact Sensitivity Maps, further escalating detection chain uncertainty. Notably, while highly complex multimodal sensory manikins exist conceptually, designs such as those proposed by Sun et al. [48] represent merely preliminary theoretical assumptions (Moderate Confidence); reliant entirely on unvalidated virtual data within the Gazebo environment, their mathematical outputs lack the physical hardware validation necessary to reliably assess metrological frequency response outside formal PFMD testing.

The exact prediction of these structural errors requires the deployment of advanced mathematical frameworks. Theoretical derivations by Herbster et al. [49] (High Confidence) provide a mathematically rigorous two-mass dynamic model (2MM) to account for material damping and stiffness, analytically surpassing the simplistic 1MM approximation mandated for normative PFMDs under ISO/TS 15066. Because the robot structure stiffness heavily outweighs the biofidelic material, the resultant contact stiffness is dictated by the series expansion $c_{res}\left(x\right)={\left(\frac{1}{c_{R},\;col\left(x\right)}+\frac{1}{c_{H}\left(x\right)}\right)}^{-1}\approx c_{H}(x)$. To accurately map the safety threshold force $F_{S}$ to the actual reaction force $F_{R}$, they apply the inverse transposition of the Jacobian matrix:

(4) $$F_{R}=F_{S}\frac{\left|f_{col}\right|}{\left|f_{TCP}\right|}$$

where $f_{TCP}=J_{P,TCP}^{-T}\left(J_{P,col}^{T}f_{col}\right)$. This framework exposes a severe unquantified uncertainty: active controller compensation during the reaction phase induces a velocity drop of up to 47%, modifying the strain-rate and physically distorting the rheological response of the PFMD damping layer. This exact formulation is complemented by Hornung et al. [50] (Moderate Confidence), who presents preliminary validation of similar dynamic frameworks. Meanwhile, empirical analyses by Clever et al. [51] (Moderate Confidence) indicate that parasitic mechanical resonances (ringing) within the supporting structure of PFMD stands may invalidate the absolute value of the isolated force peak, advocating for the application of integral quantities like power flux density, though widespread physical standardisation remains pending. Exploring alternative material architectures, Case et al. [52] (Moderate Confidence) present a soft capacitive sensor utilizing highly porous elastomers for custom biofidelic devices; however, their preliminary quantification of frequency response restricts their broader applicability. Similarly, the actively controlled motorized joint proposed by Povse et al. [53] (Moderate Confidence) transfers the physical uncertainties directly into the control loop domain of the experimental instrument without fully mitigating the mechanical filter effect. Ultimately, structural synthesis by Samarathunga et al. [54] (Moderate Confidence) outlines a preliminary approach to eliminate mechanical filtering; by physically decoupling the damping elastomers from the sensor via industrial IEPE load cells, they demonstrate how custom biofidelic instrumentation could theoretically surpass the intrinsic physical limitations of standard PFMDs, pending further robust interlaboratory validation.

### 4.2. Influence of Experimental Boundary Conditions on Energy Dissipation

The boundary conditions of the experimental setup determinatively govern the mechanisms of kinetic energy transformation and dissipation during a collision event. A strict physical differentiation exists between the state of absolutely rigid fixation (constrained collision or clamping) and an unconstrained free collision. Disregarding this explicit distinction directly leads to massive metrological errors. Early definitions, such as those formulated by Wang et al. [55] (Low/Historical Relevance), established an initial separation between these two diametrically opposed states. However, more contemporary, methodologically rigorous studies build upon this base with exact energy balances. Lachner et al. [56] (High Confidence) demonstrated that rigid fixation forces the full, unrestricted transfer of both translational and accumulated elastic energy directly into the obstacle. This scenario accurately simulates the clamping of an operator, representing the most conservative limit state where kinetic energy cannot be dissipated via kinematic rebound. Instead, it is fully absorbed by the local deformation of the tissue and the PFMD or biofidelic device, a physical phenomenon analysed in precise detail by Herbster et al. [49] (High Confidence). Further investigations of absolute clamping scenarios and associated dynamic mass distributions are presented by Sun et al. [48] (Moderate Confidence). Byner et al. [57] (Moderate Confidence) formulated a mathematical definition for kinetic energy transfer when a limb is blocked against a rigid obstacle, while Rajaei et al. [36] (Moderate Confidence) investigated the dynamics of energy transformation during absolute support by measuring deformation on the opposite side of the arm. These strictly stationary or exclusively quasi-static models constitute the core of the numerical interpolations presented by Kovinčić et al. [58] (Moderate Confidence), an analytical model subsequently expanded to encompass the spatial coverage of these conditions within the robotic workspace in their later work [59] (High Confidence). Similarly, Hüsing et al. [34] (Moderate Confidence) explicitly operated with quasi-static events representing clamping scenarios.

Executing tests exclusively on absolutely rigid stands generates unrealistic pressure and force peaks during the evaluation of transient events. Early empirical evidence of this distortion was provided by Matthias et al. [44] (Low/Historical Relevance); by implementing electromagnetic fixation on a linear guide, they demonstrated a massive escalation in detected forces when the kinematics were blocked. More recent analyses critically evaluate these limitations. Herbster et al. [60] (Moderate Confidence) rigorously analysed the physical limitations of rigid fixation, which artificially restricts natural free movement, and subsequently defined an innovative conversion method. Addressing macroscopic compliance, Han et al. [61] (High Confidence) proved on human subjects that the compliance of a relaxed human body substantially increases tolerable pain thresholds. Ignoring this compliance leads to a systematic overestimation during the prediction of biomechanical loads, a systematic error documented by Shin et al. [62] (Moderate Confidence) through simulations executing impacts against a rigid aluminium plate. Kirschner et al. [63] (Moderate Confidence) verified this physical reality, demonstrating that escalating the stiffness of the obstacle radically compresses the time integral of the collision event, while Ganglbauer et al. [64] (Moderate Confidence) attempted to numerically abstract this dynamic differentiation using computational models based on Hertzian contact theory.

To eliminate structural measurement distortion, the trajectory of research shifted toward kinematically compliant mounts. While historical attempts by Povse et al. [65] (Low/Historical Relevance) proved the technological necessity of compliant boundary conditions by implementing a motorized joint to mimic elbow damping, contemporary models offer superior metrological precision. Fischer et al. [30] (Moderate Confidence) isolated the behaviour of the measurement chain under rigid fixation, on a sliding linear guide, and on a pendulum. Their results indicate that the pendulum configuration more accurately approximates human body rebound. Samarathunga et al. [54] (Moderate Confidence) physically demonstrated the absolute necessity of measuring the pendulum’s angular deflection to objectively quantify kinetic energy transfer. Staab et al. [26] (Moderate Confidence) reached identical conclusions regarding pendulum mechanisms and their metrological superiority over conventional strain gauges. Dagalakis et al. [37] (Moderate Confidence) utilized specific mechanisms modifying substrate stiffness to simulate physiological compliance. Anthropomorphic models featuring compliant internal structures present a viable alternative, as seen in Hirata et al. [39] (Moderate Confidence) and the early head model utilized by Cordero et al. [35] (Moderate Confidence). Furthermore, Povse et al. [53] (Moderate Confidence) proposed the integration of actively controlled motorized elbows to approximate muscle impedance during transient testing.

Conversely, a distinct subset of literature exhibits methodological boundaries by focusing exclusively on the local impedance and internal dynamics of the sensor, omitting the macro-kinematics of the test stand. This highly localized approach is evident in the state-of-the-art analyses by Zimmermann et al. [7] (High Confidence) and Liu et al. [42] (High Confidence). Palmieri et al. [66] (Moderate Confidence) similarly investigated the isolated dynamic response of the instrument’s internal damping mechanism. Iki et al. [40] (Moderate Confidence) presented a biofidelic evaluation strictly of the testing skin itself, without examining overall macroscopic compliance. Additionally, St-Jean et al. [67] (High Confidence) utilized fixed boundary conditions without metrologically investigating their physical differential impact, and Ponikelský et al. [68] (Moderate Confidence) applied similarly rigid boundary assumptions. In some instances, such as Kóczi and Sárosi [69] (High Confidence), rigid fixation was explicitly selected to serve merely as a purely mechanical generator of a reference impact signal.

The inadequate definition of these boundary conditions constitutes a primary barrier to normative standardization. The interlaboratory study executed by Scibilia et al. [27] (High Confidence) empirically confirmed that even marginal differences in test stand rigidity induce severe measurement deviations. Addressing specific hardware deficits, Zhu et al. [25] (Moderate Confidence) and Samarathunga et al. [24] (High Confidence) explicitly challenged normative configurations utilizing linear guides. They argue that these mechanisms restrict kinetic energy dissipation to a single horizontal plane and simultaneously generate parasitic friction. Therefore, a comprehensive methodology strictly requires the separation of testing protocols for free transient impacts and quasi-static clamping states. Valori et al. [20] (Moderate Confidence) precisely defined this separation within their COVR methodologies, and Schneider et al. [32] (Moderate Confidence) mathematically accounted for this division within their computational framework. To bridge these metrological gaps, advanced multimodal manikins, modelled by Balletshofer et al. [70] (Moderate Confidence), are currently being developed. Schlotzhauer et al. [32] (Moderate Confidence) demonstrated the absolute importance of the virtual validation of boundary conditions across the robotic workspace. When testing transient impacts, Clever et al. [51] (Moderate Confidence) recommended the implementation of integral criteria—specifically, energy flux density—to adequately account for rebound motion. During stationary tests, applying mathematical rigidity transformation via the factor defined in ISO/PAS 5672 represents a feasible approach for standard PFMD validation, as utilized in the recent work by Caneschi et al. [38] (High Confidence). However, this specific calculation solely compensates for physical inertia, strictly neglecting the nonlinear deformation of actual biological tissue.

Boundary conditions are not determined solely by the external measurement apparatus but are heavily influenced by the internal structure of the robot itself. Steinecker et al. [71] (Moderate Confidence) established that the effective mass of a robotic manipulator is subject to spatial anisotropy. Rustler et al. [47] (Moderate Confidence) analysed how this internal spatial dependency dynamically alters the robot’s effective mass during operation. Schlotzhauer et al. [72] (Moderate Confidence) evaluated high-risk contact scenarios strictly based on the specific kinematic configuration of the robotic arm, a behaviour rigorously mapped by Švarný et al. [43] (Moderate Confidence) through comprehensive 3D maps of collision forces. Furthermore, during rigid impacts, an extremely steep shock wave occurs, frequently exceeding the signal permeability of internal detection algorithms. Huang et al. [73] (Moderate Confidence) investigated a specific back-stepping compensation method to mitigate this effect. Kirschner et al. [74] (Moderate Confidence) specifically quantified the latency and detection delay of the machine’s internal control loop when encountering solid obstacles. Kinetic energy dissipation can be purposefully modified on the manipulator side by reversing the rigidity of its outer shell, an approach rigorously investigated by Švarný et al. [75] (High Confidence). Schneider et al. [31] (Moderate Confidence) also analysed the application of an additional passive damping layer directly onto the robot.

The physical deconstruction of local boundary conditions at the contact surface level reveals further metrological deficits. High-impact empirical research by Fischer et al. [41] (High Confidence) demonstrated that if the macroscopic 3D geometry of the impactor does not correspond with the flat sensor surface of a PFMD, the resulting spatial energy redistribution generates severe parasitic deviations. Rosenstrauch and Krüger [76] (Moderate Confidence) warned that biological contact intrinsically induces demonstrable tangential and shear forces. The absence of spatial force measurement cannot be resolved even by utilizing advanced pneumatic artificial skins. Without an integrated high-resolution pressure matrix, these pneumatic biofidelic devices lack the capability to exactly localize pressure peaks. This functional deficit emerged directly from the testing of the CoboSkin sensor proposed by Heng et al. [77] (High Confidence). Therefore, to achieve the objective quantification of impacts, it is strictly unavoidable to define fully determined testing conditions. Moving away from early, outdated simplifications seen in Dombrowski et al. [78] (Low/Historical Relevance), contemporary methodologies must comprehensively incorporate external kinematic compliance, viscoelastic parameters, and dynamic inertia directly into the governing differential equations.

### 4.3. Commercial Sensor Technology Versus Innovative Biofidelic Prototypes

Detecting the spatial distribution of maximum pressure spikes represents the weakest metrological link in evaluating collaborative workspaces. While conventional Pressure and Force Measurement Devices (PFMDs) strictly adhere to established normative frameworks (ISO 5672:2023, ISO 10218-2:2025), these certified instruments exhibit severe physical artifacts. An extensive interlaboratory study executed by Scibilia et al. [27] (High Confidence) empirically demonstrated that the outputs of commercially available surface pressure sensors utilized in PFMDs—such as chemical foils or electronic matrices—exhibit extreme dispersion and unreliability during dynamic contact with non-planar cylindrical tools. The physical root of these failures lies in the inherent mechanical stiffness of conventional 2D sensory substrates. High-impact research by Fischer et al. [41] (High Confidence) demonstrated that during impacts by sharp or 3D spatial impactors, the substrate fails to conformally adapt to the topology of the contact body, inducing local bulging and generating artificial, irrelevant pressure artifacts. Zimmermann et al. [7] (High Confidence) strictly magnified this structural defect, establishing that the inertial effects of moving parts on rigid substrates generate artificial mechanical overshoots exceeding actual physical values by 10% to 25%. Furthermore, conventional 2D media systematically fail to capture the complex volumetric spatial deformations of actual biological tissue, a mechanical inadequacy empirically supported by Rajaei et al. [36] (Moderate Confidence).

From an electronic and material perspective, standard surface media lack adequate temporal resolution to capture millisecond transient phenomena. Han et al. [61] (High Confidence) identified this primary deficit, ultimately discarding data from commercial sensors due to absolute temporal unreliability. D’Antona et al. [29] (High Confidence) exactly demonstrated that both single-use chemical detectors and conventional Force Sensing Resistor (FSR) technologies fail to provide continuous real-time data and exhibit massive vulnerability to environmental fluctuations, rapidly invalidating the recorded signal. The transition from quasi-static to transient impacts induces severe accuracy drops; Kóczi and Sárosi [69] (High Confidence) mathematically documented that due to cylinder rebound and the loss of linearity, the signal correlation with the reference instrument drops into negative values. A completely disqualifying phenomenon is the limit saturation (clipping) of the measurement range in passive detectors, which Ponikelský et al. [79] (High Confidence) proved causes an immediate exhaustion of detection capacity during highly dynamic impacts. Under impacts with sharp geometries, these foils face physical destruction, a severe hardware limitation that historically led Dagalakis et al. [37] (Moderate Confidence) to propose testing through volumetric penetration into tissue simulators. Additionally, Li [80] (Moderate Confidence) argues that conventional media are physically incapable of capturing tangential friction and shear stresses. Because commercially available devices fundamentally lack the capability for reliable absolute dynamic calibration—a metrological gap documented early by Matthias et al. [44] (Low/Historical Relevance)—the scientific trajectory systematically shifts toward developing unapproved but technologically superior biofidelic instruments.

To eliminate the metrological defects associated with the stiffness of certified PFMDs, research systematically advances toward biofidelic instruments that strictly emulate the nonlinear viscoelastic impedance of human tissue. While early concepts, such as the fully compliant capacitive sensor by Case et al. [52] (Moderate Confidence), utilized porous dielectric elastomers, modern high-confidence architectures demand exact biomimesis. Iki et al. [40] (Moderate Confidence) and particularly Liu et al. [42] (High Confidence) replaced rigid substrates with sophisticated multilayer polymers that exactly replicate macroscopic human limb impedance. Addressing historic problems with pressure leakage, Kim et al. [45] (Low/Historical Relevance) constructed a monolithically 3D-printed hermetic pneumatic sensor designed explicitly for internal volumetric pressure changes. Povse et al. [53] (Moderate Confidence) advanced the field by achieving the mathematical fusion of local deformation response and macroscopic impedance through an actively controlled mechatronic prototype replicating evasive collision dynamics. To overcome the strict frequency limitations of resistive matrices, contemporary hybrid architectures implement high-speed Field-Programmable Gate Arrays (FPGA). The VSITD biofidelic prototype developed by D’Antona et al. [29] (High Confidence) efficiently combines 32 × 32 pixel pressure trace localization with exact piezoelectric measurement of absolute force amplitudes.

An independent trajectory involves the radical abandonment of the surface foil concept entirely. Advanced biomimetic systems install surface sensor matrices at defined anatomical depths to enable the separate detection of superficial and deep bone pressure, forming the structural foundation of the anatomical arm by Hirata et al. [39] (Moderate Confidence). To quantify local shear deformations and detect dynamic friction vibrationally, specific biofidelic structures, such as those evaluated by Li [80] (Moderate Confidence), embed polymeric PVDF foils directly within artificial components. Concurrently, the research focus systematically shifts away from external sensory instrumentation toward algorithmic corrections and advanced data fusion. Clever et al. [51] (Moderate Confidence) mathematically derived the dynamic contact area strictly from compression depth, utilizing robust integral criteria—specifically power flux—for safety evaluation. This trajectory is corroborated by Sun et al. [48] (Moderate Confidence), who utilized 6D force sensors and IMU units, and Kirschner et al. [46] (Moderate Confidence), who mapped the exact sensitivity thresholds of internal torque sensors embedded within manipulators. Within the domain of electronic skins, static pressure limits are systematically replaced by dynamically reconfigurable systems. Rustler et al. [47] (Moderate Confidence) developed advanced architectures that continuously calculate localized artificial pain using a mass-spring-mass model that strictly accounts for the instantaneous effective mass of the specific robotic link during contact.

### 4.4. Requirements for Standardization and Reproducibility

Achieving objective interlaboratory reproducibility of expert data constitutes the primary imperative for the safety certification of collaborative workspaces. The mere determination of biomechanical pain and force thresholds, as defined in the former ISO/TS 15066, remains functionally inadequate to guarantee physical safety. Laboratories utilizing differing instrumentation generate diametrically opposed results during physically identical impacts. This severe discrepancy was rigorously quantified by Scibilia et al. [27] (High Confidence), corroborating the methodological deviations observed by Zimmermann et al. [28] (Moderate Confidence). Consequently, the new ISO 10218-2:2025 normative framework, operating in conjunction with Annex N and the ISO/PAS 5672 specification, transforms previous advisory recommendations into exact metrological procedures. Samarathunga et al. [21] (High Confidence) and Valori et al. [20] (Moderate Confidence) explicitly articulate this shift. This systemic transformation strictly demands the standardization of the internal mechanics of measurement devices, experimental boundary conditions, and data fusion protocols.

Regarding the hardware architecture of certified pressure and force measurement devices (PFMD), contemporary high-impact research dictates that metrological directives must strictly define the tolerance fields for dynamic parameters. During millisecond transient contacts, measurement instruments inherently operate as mechanical low-pass filters. The absence of an explicit normative definition regarding the effective mass of moving parts (flywheel mass) and internal resonant frequencies induces parasitic oscillations within commercial sensors. These uncompensated oscillations artificially overestimate the impact force by up to 25%, an error magnitude confirmed by top-tier empirical studies from Samarathunga et al. [24] (High Confidence) and Zimmermann et al. [7] (High Confidence), which expand upon the initial hardware analyses of Palmieri et al. [66] (Moderate Confidence). To ensure metrological objectivity, the strict standardization of the thickness and compressive characteristics of all internal subcomponents, including damping textiles, is absolute, a requirement urged by Zhu et al. [25] (Moderate Confidence).

Furthermore, transient impact events exhibit a frequency spectrum extending to the 100 Hz boundary, as analysed by Caneschi et al. [38] (High Confidence). Therefore, the governing standards must mandate a unified sampling frequency and temporal resolution, alongside exact temporal synchronization between pressure matrices and load cells—a methodological necessity demanded by D’Antona et al. [29] (High Confidence) and Clever et al. [51] (Moderate Confidence). To eliminate sensor drift within the internal torque sensors of the manipulators, the standardization of algorithmic compensation methods is paramount, an approach synthesized by Nguyen and Case [81] (High Confidence). This must be accompanied by empirical validation utilizing certified external load cells, a procedure practically applied by Emiliani et al. [82] (High Confidence) and Ponikelský et al. [68] (Moderate Confidence).

Achieving adequate biofidelity cannot be reductionistically mapped to a unidimensional nominal spring stiffness value. While early parametric studies conceptualized simplistic approximations, Hornung et al. [50] (Moderate Confidence) decisively demonstrated this structural limitation. Current consensus mandates the application of nonlinear viscoelastic material characteristics. This guarantees that the testing phantom responds accurately across the entire temporal domain of the impact, an indispensable requirement confirmed by Liu et al. [42] (High Confidence), effectively superseding earlier mathematical propositions by Iki et al. [40] (Moderate Confidence), Rajaei et al. [36] and Hirata et al. [39] (Moderate Confidence). Specific material parameters for damping substrates must precisely correlate with exact biomechanical data (e.g., specifying a 70 Shore A foam), as stipulated by Jeanneau et al. [83] (High Confidence). Furthermore, these materials must actively eliminate the parasitic stiffness introduced by the sensor itself, an architectural approach detailed by Case et al. [52] (Moderate Confidence).

The macroscopic geometry of contact bodies represents a determinative parameter. The radius of curvature of the impactor dictates the spatial localization of stress so severely that testing with sharp geometries drastically alters the tolerable physical thresholds. Fischer et al. [41] (High Confidence) rigorously documents this phenomenon, significantly refining the initial boundary conditions explored by Shin et al. [62] (Moderate Confidence) and Schneider et al. [31] (Moderate Confidence). For an objective statistical definition of safety limits, the normative framework must adopt log-logistic models, wherein the 75th percentile yields a stable safety boundary, a high-fidelity approach established by Han et al. [61] (High Confidence). Furthermore, it is necessary to demographically regionalize tolerance thresholds concerning the specific BMI and height distributions of the local population. Park et al. [84] (High Confidence) strongly accentuates this necessity, supported by the localized demographic studies of Fischer et al. [85] (Moderate Confidence). The DITCI apparatus, developed by Dagalakis et al. [37] (Moderate Confidence), serves to unify validation procedures specifically concerning sharp-edged tools.

The stationary structural configuration of conventional PFMDs physically prevents the direct evaluation of unconstrained free impacts. Consequently, metrological legislation must require the utilization of standardized pendulum test benches. Samarathunga et al. [54] (Moderate Confidence), Fischer et al. [30] (Moderate Confidence), and Staab et al. [26] (Moderate Confidence) explicitly demonstrate the superiority of this dynamic configuration over early actively controlled mechatronic phantoms, such as those developed in obsolete testing paradigms by Povse et al. [53] (Moderate Confidence) and Povse et al. [65] (Low/Historical Relevance). When testing on rigid stands, the application of a mathematical factor for the transformation of inertial data is an absolute mandate, mathematically proven by Caneschi et al. [38] (High Confidence) and Herbster et al. [60] (Moderate Confidence). Integral metrics provide a substantially more objective physical evaluation. The scalar energy budgets proposed by Lachner et al. [56] (High Confidence) offer superior analytical advantages compared to the historical Impact Energy Density (IED) and Power Flux Density parameters referenced in older studies such as Matthias et al. [44] (Low/Historical Relevance).

Metrological directives must inevitably transition from isolated point-based measurements to comprehensive volumetric workspace testing. The implementation of Collision Force Maps (CFMs/CCFMs) and Contact Sensitivity Maps (CSMs), utilizing a strictly standardized topology of testing nodes, provides the exact analytical tool required to identify operational “blind zones”. The efficacy of this topology is demonstrated by Švarný et al. [75] (High Confidence), building upon the rigorous spatial evaluations of Švarný et al. [43] (Moderate Confidence) and Kirschner et al. [46,63,74] (Moderate Confidence). The effective mass of the manipulator fluctuates nonlinearly dependent upon its specific kinematic configuration, a dynamic variable proven by Sun et al. [48] (Moderate Confidence) and Schlotzhauer et al. [72] (Moderate Confidence), which severely challenges the static mass assumptions from earlier conceptual frameworks like Dombrowski et al. [78] (Low/Historical Relevance). The Mean Reflected Mass (MRM) metric, proposed by Steinecker et al. [71] (Moderate Confidence), introduces a justified computational simplification for this issue. The certification process must also exactly quantify the total latency of the entire braking chain. Zurlo et al. [86] (Moderate Confidence), Byner et al. [57] (Moderate Confidence), and Huang et al. [73] (Moderate Confidence) explicitly investigated this specific temporal parameter. The exact selection of measurement ranges for pressure matrices, emphasized by Ponikelský et al. [79] (High Confidence), remains a determinative factor for valid risk assessment.

Comprehensive future standardization must integrate virtual validation protocols. The mathematical data extrapolation utilizing Gaussian processes presented by Balletshofer et al. [70] (Moderate Confidence); the deployment of surrogate computational models by Kovinčić et al. [58] (Moderate Confidence) and Schneider et al. [32] (Moderate Confidence); and the dynamic impact predictions executed by Schlotzhauer et al. [33] (Moderate Confidence), Ganglbauer et al. [64] (Moderate Confidence), and Mujica et al. [87] (Moderate Confidence) collectively prove the high potential for unifying safety outputs digitally. However, to achieve normative acceptance, the directives must strictly unify the internal computational coefficients—specifically, the viscous damping differential equations and spatial mass distribution algorithms. Herbster et al. [49] (High Confidence) explicitly warns that failure to standardize these exact variables causes catastrophic divergence of outputs across differing commercial solvers, a critical validation bottleneck that supersedes the preliminary virtual testing warnings articulated by Wang et al. [55] (Low/Historical Relevance). The enduring significance of historical evaluation methods, though constrained by modern empirical metrics, is documented by Cordero et al. [35] (Moderate Confidence). Furthermore, standards must delineate exact parameters for adaptive electronic skins featuring dynamically shifting safety thresholds, as detailed by Rustler et al. [47] (Moderate Confidence) and Suita and Okawa [88] (Moderate Confidence).

## 5. Discussion

The presented discussion executes a critical synthesis of the findings extracted from the theoretical review. Subsequently, it systematically evaluates the resolution of the defined Research Questions (RQ1–RQ4). The primary objective of this chapter transcends the mere description of literature. It provides analytical insight into the metrological, hardware, and normative deficits currently limiting the objective safety evaluation of collaborative workspaces operating within the Power and Force Limiting (PFL) regime.

The subsequent subsections systematically deconstruct the physical limitations inherent to the material architecture of contemporary biofidelic instruments. Furthermore, they analyse the deterministic influence of experimental boundary conditions on kinetic energy dissipation and expose the metrological unreliability of commercial surface pressure sensors. Based on this structural critique, the text identifies existing research gaps. Consequently, it defines the unavoidable engineering parameters required for ongoing standardization processes, specifically within the context of the ISO 10218-2:2025 standard.

Beyond reflecting upon the current instrumental state-of-the-art, the discussion expands the analytical horizon toward future technological trajectories. These include proactive anti-collision systems, the integration of Artificial Intelligence, and the physiological impact of operator cognitive load. For maximum clarity, the conclusion of this chapter aggregates all isolated findings and future predictions into a comprehensive state-of-the-art overview table.

### 5.1. RQ1: Resolution Degree and Identified Research Gaps

The analysed literature provides a highly robust and consistent response to the primary premise of Research Question RQ1. The extracted studies demonstrably refute the historical assumption regarding the ideal transfer function of certified PFMDs. They exactly document that the internal material architecture operates as a nonlinear mechanical filter during high-speed transient impacts, a phenomenon analytically proven by Samarathunga et al. [24] (High Confidence). It is conclusively proven that a combination of specific physical factors dictates the overall frequency response of the measurement chain. These factors include the inertial forces of moving masses, the physical saturation of steel springs observed, for instance, in the prototype by D’Antona et al. [29] (High Confidence), and the imperfect viscoelastic energy dissipation within the covering elastomers.

The literature also successfully deconstructed severe mechanical anomalies. These include parasitic structural oscillations (ringing) described by Clever et al. [51] (Moderate Confidence), mechanical bounce-back detected by Staab et al. [26] (Moderate Confidence), and pneumatic latencies identified by Kim et al. [45] (Low/Historical Relevance). These anomalies introduce massive amplitude and phase distortions directly into the evaluation process. In this regard, the physical mechanism generating these metrological uncertainties is theoretically and empirically well documented by contemporary high-impact research.

Identified Deficits and Research Gaps: Despite a profound understanding of the physical limits inherent to existing hardware, current research regarding transient impact detection exhibits several significant gaps. These deficits limit a comprehensive metrological resolution of the problem:

Absence of rigorous uncertainty models (GUM) for dynamic events: The majority of comparative and interlaboratory studies empirically confirm an enormous variability in measured force peaks, frequently in the order of hundreds of Newtons, as demonstrated by Zimmermann et al. [28] (Moderate Confidence) and Scibilia et al. [27] (High Confidence). However, the literature chronically lacks the application of a rigorous mathematical apparatus based on the GUM standard (Guide to the Expression of Uncertainty in Measurement) specifically adapted for millisecond transient phenomena. While static and quasi-static sensor uncertainties are relatively well documented, the exact analytical formulation of cascaded error propagation during dynamic impacts remains unresolved. This mathematical formulation must encompass the entire measurement chain, spanning from the nonlinear deformation of the elastomer to the A/D converter.

Deficit of inverse methods for signal reconstruction: Existing publications detail how the material architecture dampens steep force gradients and acts as a mechanical low-pass filter. Nevertheless, proposals for robust inverse deconvolution algorithms remain largely absent. If the mechanical impedance (transfer function) of a specific instrument is known, it should be theoretically possible to retroactively reconstruct the actual surface impact force profile directly from the distorted recorded signal. However, current research is restricted either to the simple statement of the error or to bypassing the problem by transitioning toward integral quantities (transferred energy/power). Clever et al. [51] (Moderate Confidence) proposed this specific transition. Consequently, the literature fails to offer a direct mathematical correction for the raw force signal.

Technological gap between validation and the implementation of 3D biofidelity: Theoretical studies precisely define hyper-viscoelastic models of human tissue, as evidenced by Rajaei et al. [36] (Moderate Confidence) and highlight the inadequacy of the 1D normative approximation. Despite this, the actual implementation of these findings into certified testing practice severely lags. Innovative approaches face severe validation limits. These include multimodal manikins tested within the Gazebo environment by Sun et al. [48] (Moderate Confidence), soft capacitive skins developed by Case et al. [51] (Moderate Confidence), and actively controlled motorized joints designed by Povse et al. [53] (Moderate Confidence). Currently, these architectures are validated predominantly within isolated laboratory conditions or exclusively within purely simulated environments. There is an absolute lack of independent empirical studies subjecting these novel material architectures to strict frequency limit testing during actual high-speed impacts against industrial robots operating within the PFL regime.

Inadequate quantification of sensor interaction with boundary conditions: The literature convincingly documents the severe impact of asymmetric collisions and off-axis forces on data dispersion. Fischer et al. [41] (High Confidence) and Valori et al. [20] (Moderate Confidence) specifically accentuate this phenomenon. However, there is a near-total absence of parametric studies fully quantifying the dependence of the sensor’s metrological error on the complex 3D geometry of the impactor (tool) combined with the variable hardness of supplementary damping. Schneider et al. [31] (Moderate Confidence) provided only a partial foundation for this analysis. Currently, these variables are investigated primarily in isolation, rather than being approached as a multidimensional problem within nonlinear continuum mechanics.

### 5.2. RQ2: Resolution Degree and Identified Research Gaps

The analysed literature provides a highly robust and physically consistent response to Research Question RQ2. The extracted studies irrefutably demonstrate that experimental boundary conditions do not constitute merely a passive testing background. Instead, they represent the primary deterministic factor governing the transformation of kinetic energy. The research consensus clearly defines that the absolutely rigid fixation of the sensor artificially eliminates macroscopic kinematic compliance. Consequently, this configuration forces the system to absorb all mechanical work exclusively through the local deformation of the covering layers and the sensor itself. Lachner et al. [56] (High Confidence) and Herbster et al. [49] (High Confidence) mathematically document this physical phenomenon. The direct consequence of this stationary mounting is an extreme systematic overestimation of maximum pressure and force peaks during the simulation of unconstrained free impacts. Matthias et al. [44] (Low/Historical Relevance) empirically verified this specific metrological distortion.

The literature successfully deconstructed this signal amplitude distortion through the application of pendulum and compliant testing systems. These specific architectures proved that kinematic deflection (both translational and rotational) absorbs a significant fraction of the transferred momentum. Han et al. [62] (High Confidence) demonstrated this absorption physically using human subjects, while Samarathunga et al. [54] (Moderate Confidence) achieved analogous results utilizing testing pendulums. This mechanical compliance induces an enormous prolongation of the impact time integral, leading to a demonstrable reduction in the detected force extremes.

Identified Deficits and Research Gaps: Although contemporary literature thoroughly documents the physical dichotomy between constrained clamping and unconstrained free impacts—a distinction explicitly defined by Wang et al. [55] (Low/Historical Relevance)—the practical metrological integration of these findings exhibits several severe gaps.

Absence of multiaxial detection within compliant setups: The literature exactly proves that biological contact is not a unidimensional phenomenon. Significant tangential and shear forces emerge during physical compression, a fact strictly warned against by Rosenstrauch and Krüger [76] (Moderate Confidence). Despite this, the overwhelming majority of experimental apparatuses, including pendulum setups, rely exclusively on 1D strain gauges or pressure sensors. There is an absolute lack of research rigorously comparing the influence of boundary conditions on the complex 3D stress tensor. Fischer et al. [41] (High Confidence) explicitly highlighted the spatial artifacts generated by 1D sensors, yet this physical deficit remains unresolved. The inclusion of shear forces remains fundamentally required for the objective definition of soft tissue injury limits.

Deficit of dynamic data fusion between the robot and the sensor: Existing publications convincingly demonstrate that the effective mass of the robotic manipulator is highly anisotropic. This parameter fluctuates dynamically dependent upon the specific kinematic pose, as mathematically defined by Steinecker et al. [71] (Moderate Confidence) and Rustler et al. [47] (Moderate Confidence). Nevertheless, the testing devices (PFMDs) continue to operate as isolated “black-box” systems. The literature completely lacks proposals for real-time mechatronic data fusion. Within such an architecture, the robot’s internal control system would continuously transmit its instantaneous effective mass and directional velocity vector directly into the diagnostic instrument. This integration would enable the dynamic algorithmic correction of the detected pressure peaks directly during the execution of the safety audit.

Metrological vacuum regarding sequential collision events: Existing methodologies, such as the COVR protocols established by Valori et al. [20] (Moderate Confidence), strictly polarize safety testing into either unconstrained free impacts or quasi-static clamping. However, actual collisions within unstructured industrial environments frequently manifest as sequential events (so-called “push-to-clamp” scenarios). In these instances, a free impact transitions continuously into a state of absolute rigidity the moment the limb is forced against a solid obstacle. Contemporary literature lacks continuous mathematical and experimental models capable of fluidly describing the nonlinear jump in mechanical impedance during this transitional state. Consequently, it remains impossible to exactly quantify the resulting pressure peak without artificially dividing the experiment into two isolated measurements.

Insufficient replication of boundary conditions for pneumatic sensors: Although soft and pneumatic artificial skins provide excellent macroscopic compliance, their physical response to specific boundary conditions remains highly nonlinear due to internal gas thermodynamics. Research has not yet resolved the inherent technological paradox evident, for example, in the testing of the CoboSkin sensor proposed by Heng et al. [77] (High Confidence). How to ensure the high-frequency sampling of a pressure matrix integrated upon a highly deformational substrate without the internal gas compression acting as a parasitic low-pass filter remains unresolved. This pneumatic latency fundamentally obstructs the exact spatial localization of the transient pressure peaks.

### 5.3. RQ3: Resolution Degree and Identified Research Gaps

The analysed literature provides a definitive and highly critical response to Research Question RQ3. The extracted studies empirically and theoretically disqualify existing commercial surface pressure sensors (specifically, chemical foils and resistive matrices). These conventional devices fundamentally fail as reliable metrological instruments for evaluating transient impacts, a metrological deficit extensively documented by Scibilia et al. [27] (High Confidence).

Research exactly demonstrated that these conventional technologies physically fail due to their inherent mechanical stiffness. This physical rigidity generates artificial bulging when subjected to complex impactor geometries, a structural artifact proven by Fischer et al. [41] (High Confidence). Furthermore, these sensors suffer from severe electronic deficits. Primarily, they exhibit immediate limit saturation (clipping) of their measurement range, a phenomenon explicitly observed by Ponikelský et al. [79] (High Confidence). Additionally, they demonstrate completely inadequate temporal resolution. This specific failure forced Han et al. [61] (High Confidence) to discard data from these sensors entirely. As an adequate technological response, the scientific community successfully formulated a broad spectrum of innovative alternatives. These architectures range from fully conformal capacitive and hermetic pneumatic sensors, developed by Case et al. [52] (Moderate Confidence) and Kim et al. [45] (Low/Historical Relevance), to sophisticated biomimetic arms featuring actively motorized impedance, presented by Povse et al. [53] (Moderate Confidence). Finally, the field exhibits a radical paradigm shift toward integral criteria and proprioceptive data fusion.

Identified Deficits and Research Gaps: Although the literature extensively details the development of alternative prototypes and the structural deconstruction of commercial foil defects, the transition of these innovations into applied industrial practice remains blocked by several severe scientific gaps:

The gap between laboratory validation and industrial scalability: Non-commercial prototypes demonstrate excellent biofidelity during strictly controlled experiments. These architectures include 3D-printed pneumatic modules by Kim et al. [45] (Low/Historical Relevance), Maxwell polymeric structures applied by Iki et al. [40] (Moderate Confidence), and actively controlled mechatronic joints by Povse et al. [53] (Moderate Confidence). Nevertheless, existing publications almost completely ignore the fundamental question of their long-term mechanical durability and stability (MTBF). There is an absolute absence of empirical studies testing the physical fatigue of these compliant viscoelastic materials following thousands of impact cycles. The evaluation of their resistance to industrial environmental degradation is similarly missing. This deficit currently prevents their structural transformation into certified metrological apparatuses.

Absence of a normative framework for integral and multimodal criteria: Researchers are systematically shifting away from unreliable pressure foils toward advanced data fusion (e.g., IMU units, 6D force sensors), as investigated by Sun et al. [48] (Moderate Confidence). A similar trajectory involves the mathematical derivation of power flux density, proposed by Clever et al. [52] (Moderate Confidence). This transition represents an exact physical advancement. However, this trend collides severely with a standardization vacuum. The current normative framework (ISO 10218-2:2025) strictly mandates the physical evaluation of surface pressure and maximum force. The literature lacks robust mathematical conversion models capable of rigorously correlating these novel biofidelic metrics, such as the dynamically calculated “artificial pain” developed by Rustler et al. [47] (Moderate Confidence), with existing medical limits for biomechanical injury.

Inadequate quantification of computational latency and industrial deployment barriers: The hardware delays of commercial sensors are documented thoroughly. Conversely, deeper analytics regarding the total cascaded software processing latency are absent for innovative reconfigurable systems and hybrid architectures. This specific deficit applies to the VSITD prototype by D’Antona et al. [29] (High Confidence) and the e-skin developed by Rustler et al. [47] (Moderate Confidence). The continuous calculation of dynamic sensitivity thresholds based on the instantaneous kinematic matrix of the robot demands extreme computational power. To provide a concrete engineering perspective, future research must explicitly deconstruct this computational bottleneck into specific latency sources. In actual industrial deployment, the total safety response time constitutes a cumulative cascaded delay encompassing AI inference time (data processing within the algorithmic layer), PLC scan cycles (execution time of the programmable logic controller), communication bus delays (deterministic data transfer across networks), and the ultimate safety-system response time (electromechanical engagement of mechanical brakes). The severe impact of this total cascaded latency on the timely initiation of emergency braking during millisecond transient impacts remains heavily underestimated across contemporary publications, consequently requiring extensive physical validation before these predictive models can achieve certifiable industrial deployment.

Ignorance of multiaxial trauma in novel prototypes: Fischer et al. [41] (High Confidence) exactly deconstructed the physical failure of 2D foils during the detection of spatial artifacts. Although this finding initiated the development of conformal artificial skins, most innovative laboratory models still operate strictly by optimizing the deformation response solely for the normal force component. Li [80] (Moderate Confidence) presented isolated studies applying PVDF foils for the vibrational analysis of friction. Excluding these rare exceptions, the literature fundamentally lacks functional prototypes capable of continuously mapping tangential and shear stresses across a large surface area with high spatial resolution. These specific multidirectional force vectors remain deterministic for the onset of actual skin lacerations.

### 5.4. RQ4: Resolution Degree and Identified Research Gaps

The analysed literature provides a highly comprehensive and mathematically rigorous response to Research Question RQ4. The scientific consensus deconstructed the inadequacy of the previous normative framework. Furthermore, it precisely defined the metrological imperatives required to achieve interlaboratory consensus, an analytical parameter notably absent in historical testing, as rigorously quantified by Zimmermann et al. [28] (Moderate Confidence) and Scibilia et al. [27] (High Confidence). The extracted texts unambiguously demonstrate that standardization must not remain restricted to isolated parameters. Instead, it must comprehensively cover the entire data acquisition chain. Publications successfully formulated the strict necessity to unify internal mechanics. This specifically includes the inertial mass of moving parts (flywheel mass) and resonant frequencies, as defined by Samarathunga et al. [24] (High Confidence). Furthermore, the unification must encompass electronic architectures, mandating sampling rates exceeding 1 kHz and the precise synchronization of data streams, as demanded by Clever et al. [51] (Moderate Confidence). Finally, it requires the strict definition of material biofidelity regarding nonlinear viscoelastic properties and macro-geometry. Fischer et al. [41] (High Confidence) explicitly warned against the metrological defects arising from the neglect of these geometric parameters. Moreover, the response to RQ4 is heavily enriched by a paradigm shift toward volumetric spatial validation. Examples include the implementation of CFM maps by Švarný et al. [43] (Moderate Confidence) and the MRM parameter introduced by Steinecker et al. [71] (Moderate Confidence). Consequently, contemporary research exhaustively describes the physical mechanism generating metrological discrepancies and lists the exact parameters required for their elimination.

Identified Deficits and Research Gaps: Although the literature explicitly enumerates the specific parameters requiring normalization within the context of ISO 10218-2, the process of transforming these variables into actual certification practice reveals several severe gaps and unresolved technological paradoxes:

Absence of normative algorithmization for virtual validation: Research demonstrably proves the necessity of transitioning toward predictive computational models. This includes the utilization of Gaussian processes proposed by Balletshofer et al. [70] (Moderate Confidence) or surrogate models developed by Kovinčić et al. [59] (High Confidence). However, the literature completely lacks a unified consensus regarding which specific mathematical apparatus will achieve legislative acceptance. An exact methodology for unifying the differential equation solvers utilized within commercial software environments is completely absent. The safety standards must strictly prescribe the permitted mathematical models for deriving effective mass and calculating viscous damping, an imperative demanded by Herbster et al. [49] (High Confidence). Until this occurs, virtual validation will continue to generate statistically divergent results across different testing laboratories.

Metrological vacuum in evaluating internal software latency: Authors repeatedly demonstrate that actual physical safety is strictly determined by the total braking distance and the processing latency of internal Generalized Momentum Observers (GMO). Huang et al. [73] (Moderate Confidence) and Byner et al. [57] (Moderate Confidence) explicitly investigated these temporal variables. However, testing laboratories continuously encounter the proprietary, closed “black-box” architectures of the robotic control systems designed by manufacturers. The literature lacks proposals for independent, hardware-agnostic testing protocols. Such protocols are required to objectively measure the exact temporal delay between physical impact, software detection, and mechanical deceleration across systems from various vendors, strictly without requiring access to the proprietary source code.

Disproportion between demographic reality and hardware unificationTheoretical studies unambiguously prove that injury thresholds fluctuate extremely depending on BMI, age, and the specific anatomical region. Park et al. [84] (High Confidence) and Fischer et al. [85] (Moderate Confidence) empirically documented this biological variance. Conversely, guaranteeing interlaboratory reproducibility mandates the maximal unification of the PFMD hardware. This dictates a severe restriction on the permitted number of physical testing phantoms. Contemporary research has not yet provided a pragmatic engineering resolution to this metrological paradox. There is no existing design for a universal “worst-case” biofidelic material. Such a material must metrologically cover extreme demographic variables without degrading the certification process into the endless physical testing of dozens of distinct compression elements.

Insufficient experimental validation of conversion coefficients: The application of mathematical conversion factors (e.g., the specific multiplier utilized to recalculate rigid measurements into unconstrained free impact equivalents) is postulated as an unavoidable standardization tool. Caneschi et al. [38] (High Confidence) and Herbster et al. [60] (Moderate Confidence) mathematically applied these multipliers. Nevertheless, the literature completely lacks extensive empirical studies validating these purely mathematical transformers against complex, asymmetrical 3D impacts featuring prominent tangential friction. A severe risk persists that these simplified, unidimensional multipliers physically fail during oblique impacts involving sharp geometric edges. Consequently, in industrial practice, this mathematical simplification can easily generate false-positive outcomes during safety audits.

### 5.5. Future Research Trajectories: From Reactive Mitigation to Predictive Prevention

The normative framework governing collaborative robotics is undergoing a gradual evolution. Geiger et al. [89] executed a comprehensive mapping of the relevant technical standards within collaborative robotics up to early 2025. Their publication details the individual robotics standards immediately prior to the integration of the ISO/TS 15066 specification into the revised ISO 10218-2:2025 standard. As part of this recent update, the “safety-rated monitored stop” collaboration method has been removed from the list of collaboration methods and is now solely a safety feature of the robot. The reclassification of the “safety-rated monitored stop” from a direct collaboration method to a general safety feature of the robot (within the ISO 10218-2:2025 revision) represents a minor, yet discernible, regulatory step. While this structural change theoretically permits more flexible application designs, industrial practice remains rigidly bound by deterministic safety requirements. Currently, true proactive collision prevention lacks explicit standardization. Industrial implementations strictly rely on certified reactive systems (e.g., PLCs, safety scanners) because proactive models, often based on probabilistic AI or complex sensor fusion, cannot yet guarantee the Required Performance Level (PLr) or meet hardware fault tolerance metrics defined by ISO 13849-1:2023 [90]. Consequently, while contemporary academic research increasingly focuses on the unification of the PFL and SSM regimes into a proactive hybrid mode, an extensive gap persists between these laboratory concepts and certifiable industrial deployment.

Lucci et al. [91] mathematically formalized this hybrid concept. The authors proposed an advanced optimization model that enables the manipulator to maintain higher operational velocities in close proximity to the human operator. Their derived equation strictly guarantees sufficient braking capacity. Consequently, any physical contact at the point of impact will not exceed the designated PFL biomechanical limits. The proposed algorithm iteratively scales the planned trajectory and incorporates a dynamically variable inertia matrix. This approach successfully replaces previous inefficient conservative estimates. Manzardo et al. [92] critically expanded upon this model. They identified the conventionally utilized fixed stopping time as a highly restrictive parameter. Accordingly, they replaced it with a dynamically calculated minimum deceleration time. This novel calculation utilizes inverse dynamics, and the limiting joint torques of the actuators to accurately define the spatial volume traversed by the robot during braking. If this braking volume avoids intersection with the predicted operator space, the system prevents a forced emergency stop. This predictive logic significantly reduces operational downtime and enhances kinematic smoothness without compromising physical safety.

The practical industrial implementation of these proactive algorithms requires the integration of electronic skins (e-skins). These sophisticated interfaces fuse passive impact energy absorption with active proximity detection. Metrologically, these systems can be deployed globally across the entire kinematic chain or localized to specific segments. Regarding full-body spatial coverage, Niquet et al. [93] introduced a multimodal textile fusing capacitive, ultrasonic, and Time-of-Flight (ToF) sensors. This specific design primarily targets low-cost applications within small and medium-sized enterprises. The cascaded control architecture achieves proximity detection up to a distance of four meters. Nevertheless, the system exhibits unacceptable communication latency and severe sensor interference. These hardware deficits induce oscillatory deceleration profiles, ultimately causing extreme mechanical wear on the robotic joint actuators. Zhou et al. [94] developed a significantly more advanced architecture through the modular TacSuit system. This prototype integrates 3D-printed hexagonal cells equipped with MEMS pressure sensors, proximity detectors, and inertial measurement units. The architecture resolves inherent data redundancy issues through an event-driven topology, successfully reducing the communication bus load by 95%. Furthermore, the algorithm converts distance and pressure data into a virtual interaction force vector. This mathematical transformation allows the manipulator to execute active avoidance maneuvers, mitigating contact forces substantially faster than a conventional emergency stop. Despite its high biofidelity, the serial cell connection introduces a critical single-point-of-failure vulnerability. Furthermore, the architecture remains limited by prohibitive manufacturing costs.

Localized instrumentation of the end-effector provides a viable alternative to full-body sensorization. Tsuji and Kohama [95] constructed a hybrid sensor utilizing a self-capacitance principle. Within this design, an ethylene-propylene rubber (EPR) layer functions simultaneously as a dielectric medium and a passive mechanical damper. The embedded microcontroller continuously alternates between active proximity detection and physical pressure measurement. The experimentally optimized 10 mm rubber thickness effectively dissipates transient impact energy. Although architecture represents a computationally elegant and unified solution, the physical principle of capacitive measurement imposes severe operational limitations. The system demonstrates excellent detection capabilities for conductive human bodies at distances up to 120 mm. Conversely, it remains practically incapable of detecting dielectric materials, including plastic crates or composite structural panels. Consequently, during a collision with such objects in a heterogeneous industrial environment, the robot relies entirely on the passive mechanical damper. This singular reliance proves fundamentally insufficient for fully autonomous and safe collaborative operations.

The hypothetical integration of mobile humanoid robots would likely necessitate a structural reassessment of current e-skin topologies. Unlike static manipulators, bipedal humanoids exhibit theoretical unconstrained spatial mobility, kinematic redundancy, and a highly variable interaction space dependent on relative dynamic heights. Adapting e-skins for such complex multi-DoF structures might theoretically require anatomically compliant sensory networks capable of withstanding multi-axial stretching without generating strain-induced signal artifacts. Furthermore, future computational architectures would presumably need to continuously recalculate tactile collision vectors relative to a fluctuating Center of Mass (CoM) to probabilistically differentiate stochastic external impacts from intentional physical interactions or kinematic self-collisions.

The deployment of Artificial Intelligence (AI) algorithms and computer vision systems for contactless collision avoidance inevitably complements this hardware layer. Spatial data fusion from RGB-D cameras or LiDAR sensors enables the continuous algorithmic approximation of human kinematics. For instance, Calcagni et al. [96] utilize depth sensor fusion to model obstacles as ellipsoids. Subsequently, they apply the Artificial Potential Field (APF) method within the null space of the Jacobian matrix of a seven-axis manipulator to execute evasive maneuvers. Wang et al. [97] introduced the HCRI interface for ROS2 as an alternative to computationally intensive voxel mapping. This architecture employs neural networks to extract the SMPL biomechanical model from a standard RGB image, effectively reducing the human body to simplified geometric primitives. Although this solution radically reduces the planning loop latency, geometric inaccuracies necessitate a 20% artificial volumetric expansion of the safety envelopes. Furthermore, the system remains severely limited by optical occlusions and the inherent drift of inertial measurement units.

Scimmi et al. [98] adopted a fundamentally different approach to spatial detection. They deployed the OptiTrack infrared marker system within their experimental setup and controlled evasive maneuvers via external distributed potential fields. The system successfully isolates the end-effector trajectory from elbow evasions, effectively reducing the 6-DOF problem to a 3-DOF task. However, the presented configuration functions primarily as an early laboratory prototype. Successful integration into unstructured industrial operations strictly demands hard real-time latencies. Therefore, future research must eliminate the operational dependence on passive markers. Moreover, it must demonstrably increase the frequency of the asynchronous communication loop, which peaked at only 62.5 Hz in this initial experimental configuration.

The transition from simple reactive detection toward advanced prediction represents a higher evolutionary stage. Cheng and Tomizuka [99] proposed a hierarchical “detect and predict” framework. This architecture classifies human intent utilizing Long Short-Term Memory (LSTM) neural networks. The prediction of hand trajectories via a sigma-lognormal model represents a primary innovation of this approach. This mathematical model rigorously derives from the neurological foundations of human motion control. Through algorithmic online adaptation to the operator’s specific biomechanical style, this model demonstrates superior accuracy compared to conventional minimum jerk models. The average prediction error effectively falls below the 25 mm threshold. Consequently, Artificial Intelligence endows robotic systems with the capability of dynamic, real-time trajectory replanning. This proactive capability minimizes the probability of physical contact without enforcing a hard operational stop of the production line.

Despite the advancements presented in these predictive and vision-based architectures, current proactive AI systems exhibit a severe vulnerability regarding computational latency and non-deterministic execution times. Industrial safety standards for Power and Force Limiting applications require hard real-time processing guaranteed by fail-safe Programmable Logic Controllers (PLCs) and deterministic communication buses (e.g., PROFIsafe, FSoE). Conversely, current AI implementations heavily rely on high-level frameworks like ROS2 [96], standard TCP/IP communication [97], or stochastic nonlinear optimizations [98], which inherently lack Worst-Case Execution Time (WCET) guarantees. This discrepancy is particularly evident in recent approaches utilizing cloud-based processing or computationally heavy architectures. For instance, Liu et al. [100] proposed an architecture integrating an open-architecture ROS computer connected to the OpenAI GPT-4 model. This system operates with an RGB-D camera sampling rate of only 30 Hz, introducing significant hardware latency before algorithmic processing even begins. Similarly, Makris et al. [101] implemented an AI-based vision system that uploads image data to the IBM Watson cloud service for classification. The authors explicitly describe this collision detection system as operating merely in “near real-time”. From a strict industrial safety perspective, such latencies are unacceptable. During an inference and communication delay spanning tens or hundreds of milliseconds, a manipulator can easily breach the permitted biomechanical pressure and force limits before a halt command is executed. Consequently, AI and vision systems currently function exclusively as asynchronous, macro-level planning layers. Until deterministic Edge-AI accelerators are integrated directly into low-latency safety buses, physical and reactive elements remain irreplaceable. They establish the primary, hardware-bound safety loop that instantaneously reacts to physical contact whenever the proactive layer reacts too slowly or fails entirely.

Augmented, Mixed, and Virtual Reality (AR/MR/VR) technologies significantly enhance the cognitive interface between the human and the machine. Immersive interfaces enable the direct projection of intended manipulator trajectories and safety zones into the operator’s field of view. This visualization radically improves spatial orientation and mitigates dangerous path intersections caused by human inattention. Gruenefeld et al. [102] empirically verified this premise by testing diverse modalities for visualizing robotic intent through an AR headset. Their comparative analysis conclusively demonstrated that the volumetric projection of the robotic arm’s future spatial path is significantly more effective than the conventional visualization of a 1D trajectory. This volumetric approach enabled operators to work in closer proximity to the manipulator without initiating unintended safety stops. Furthermore, it demonstrably minimized the necessity for physical head rotations. However, immersive interfaces function beyond mere passive displays. Ong et al. [103] demonstrated the potential of AR for intuitive robotic programming. In this paradigm, the operator defines trajectory waypoints directly within the physical workspace. Utilizing spatial scanning, the system autonomously generates collision-free trajectories. This spatial programming reduces the overall time expenditure and eliminates the tedious manual operation of conventional control teach pendants.

The concept of Digital Twins demonstrates an even deeper integration of proactive safety elements. Subramanian et al. [104] implemented an MR interface acting as a full-fledged safety sensor. This approach successfully overcomes the inherent problems of blind spots and optical occlusions plaguing physical sensors. By continuously tracking the operator’s head position, the system transforms human kinematics into simplified virtual collision primitives within a computational simulation engine. The engine calculates the shortest absolute distance between the virtual human model and the robotic avatar in real time. This exclusively virtual data serves as a direct, exact input for the SSM algorithm, which dynamically regulates the physical speed of the real-world manipulator. Paniti et al. [105] subsequently presented a paradigm shift from pure visual mitigation to multimodal prediction. Their VR architecture synchronizes the optical tracking of human kinematics with a forward simulation of the active robotic program. If the algorithm detects an impending trajectory intersection within the virtual environment, it immediately initiates a haptic response via a wearable vibrational device. Consequently, the operator receives an advanced warning. This latency allows for the cognitive correction of movement long before the metrological or anti-collision instrumentation would be forced to resolve a physical impact.

### 5.6. Demographic Anisotropy and the Impact of Cognitive Overload

The metrological inadequacy of current standards is critically evident in the artificial unification of biological subjects. Existing biomechanical limits operate with a general threshold for pain and tissue damage. This singular threshold is applied uniformly across the entire population. This rigid approach completely ignores substantial variations stemming from gender, age, Body Mass Index (BMI), muscle tone, and skin elasticity. Behrens et al. [106] exactly demonstrated this physical deficit. By measuring pain thresholds in 112 subjects, the authors identified gender as a relevant statistical covariate. This specific variable demonstrably alters the physical tolerance threshold across multiple anatomical structures. The construction of log-logistic distribution models confirms a fundamental requirement for future normative revisions. To objectively guarantee safety, it is essential to initiate the pluralization of limits. Furthermore, future standards must introduce stratified safety models that dynamically scale the maximum permissible forces according to specific demographic parameters.

A hypothetical resolution to this metrological discrepancy could involve the adoption of a strict “worst-case” boundary approach. Within a theoretical framework, attempting to physically map the entire demographic variance via multiple biofidelic surrogates appears unfeasible for standardized industrial practices. Instead, a viable compromise might be found in defining a singular, highly conservative sensor interface conceptually calibrated to the lowest biomechanical tolerance percentiles.

Implementing such a theoretical simplification could potentially streamline and accelerate the certification process, as safety validation would be restricted to a single, strict baseline. However, the adoption of a worst-case biofidelic surrogate would inherently shift the burden of physical compliance onto the application design itself. To satisfy these conservative testing thresholds, integrators would likely need to prioritize rigorous application optimizations. This might manifest as a systematic reduction in allowable kinematic velocities or a necessity to integrate supplementary energy-absorbing macrostructures (e.g., compliant soft skins) directly onto the robotic manipulator. Consequently, this theoretical paradigm suggests a direct metrological trade-off: maximizing demographic safety and simplifying the testing methodology at the probable expense of the collaborative system’s overall kinematic efficiency.

Furthermore, normative validation processes assume fully determined human behaviour. Consequently, they are completely abstract from stochastic phenomena associated with cognitive ergonomics and psychological stress. HRC applications generate a highly specific type of mental load. Pollak et al. [107] empirically demonstrated that working alongside fully autonomous robots induces a significant increase in heart rate and a subsequent loss of perceived personal control over the machine. Cumulative fatigue and unaccounted psychological stress demonstrably degrade operator attention. This cognitive degradation directly escalates the frequency of unpredictable, erroneous movements. Liu et al. [108] reinforced this physiological parameter. The authors highlighted the necessity of implementing a “psychological safety field” into robotic trajectory planning. An algorithmic path optimization incorporating the operator’s electrodermal and electromyographic (EMG) activity measurably reduced the physiological stress response in 87% of the tested subjects. Conventional normative models completely overlook this critical physiological variable.

Current attempts to incorporate the operator’s cognitive state into the active control loop encounter severe hardware and algorithmic limits. Campagna et al. [109] demonstrated that utilizing convolutional neural networks to assess the operator’s confidence level via facial expressions yields an accuracy strictly below 80%. Therefore, isolated reliance on a single visual sensor remains fundamentally inadequate. The escalation of stochastic kinematic deviations resulting from cognitive overload directly increases the risk of impacts occurring outside certified zones. Consequently, collisions impact unexamined anatomical areas that were not metrologically mapped during the initial safety audit. The elevated error rate of a fatigued operator thus induces high-risk scenarios where local biomechanical limits may be fatally exceeded. Therefore, holistic safety assessments must not remain exclusively limited to the physical impedance of the hardware. Future paradigms must necessarily incorporate a fusion of multimodal sensors for the continuous monitoring of psychophysiological fluctuations and the cognitive error rates of the human operator.

### 5.7. Synthesis of Research Gaps and Trajectories for Future Development

The concluding section of this discussion aggregates the identified metrological, hardware, and methodological deficits into a comprehensive overview (Table 2). The primary objective of the following table is to provide a structured reference framework. This framework clearly synthesizes the state-of-the-art while explicitly defining trajectories for subsequent research endeavours. The exact deconstruction of current technological limitations offers researchers and application engineers a precise guide for selecting highly relevant investigative topics. Resolving these identified deficits represents an absolute prerequisite for successful implementation within upcoming revisions of international safety standards.

## 6. Conclusions

This review article has provided a comprehensive synthesis of the technological development and metrological state of certified measurement devices (PFMDs) and biofidelic prototypes within the context of collaborative robotics. Through a rigorous literature analysis, the physical limitations of existing architectures were systematically deconstructed. Furthermore, the methodological deficits that have previously hindered full interlaboratory data reproducibility during safety validation in the Power and Force Limiting (PFL) regime were explicitly defined.

A quantitative screening of publication activity revealed an exact divergence between macroscopic and microscopic trends within the field. Although the global popularity of collaborative robotics and HRI/HRC issues steadily grows each year, the narrowly profiled topic of pHRI metrology and the evaluation of PFL applications exhibits a slight downward trajectory following its peak in 2021. This publication peak represented the five-year technological latency required for the absorption and experimental reflection of the 2016 ISO/TS 15066 technical specification. The current stabilization at approximately ten specialized studies annually empirically demonstrates that the research focus of collaborative robotics is gradually shifting from the reactive measurement of mechanical impacts toward alternative domains.

The longitudinal distribution of the 68 finally selected publications confirms the dominance of RQ4 (64 publications). This fact demonstrates the urgent need within the scientific community to unify safety certification processes. The high saturation of RQ2 (51 publications) reflects a growing awareness regarding the deterministic influence of experimental boundary conditions and kinetic energy dissipation within test stands on measurement validity. Research question RQ1 (35 publications) constitutes a stable centre of publication interest. This consistency proves the permanent necessity to exactly deconstruct the physical and material limits of the biofidelic sensors themselves. Conversely, the relatively low frequency of studies critically analysing commercial sensor technology (RQ3—21 publications) reveals a persistent reliance on closed “black-box” solutions, significantly lacking profound hardware critique. Regarding the technological categories, a primary shift is evident. The focus has moved from the biomechanical characterization of the human body, which is now considered sufficiently mapped—toward advanced numerical modeling and the development of surrogate models. This trend indicates a systematic effort to reduce economically demanding physical tests through the implementation of digital twins.

The formulated research questions were answered with a high degree of exactness. RQ1 and RQ2 elucidated the mechanisms of nonlinear impact filtering and the influence of fixation rigidity on the amplitude distortion of force peaks. RQ3 identified the technological limits of pressure foils, specifically their susceptibility to signal saturation and insufficient temporal resolution. Simultaneously, RQ4 defined uncompromising metrological parameters. These range from the standardization of the inertial masses of moving parts (flywheel mass) to the strict requirement of sampling frequencies exceeding 1 kHz. These parameters represent an absolute prerequisite for achieving objective interlaboratory consensus.

In the upcoming years, a radical paradigm shift from reactive mitigation toward predictive collision prevention is anticipated. Future research trajectories point toward the deep integration of electronic skins equipped with active proximity sensing. Furthermore, they involve the deployment of Artificial Intelligence algorithms for human intent recognition and the utilization of immersive technologies (AR/VR/MR) to visualize the workspace, robotic trajectories, and hazardous interactions. It also proves strictly necessary to pluralize biomechanical limits to account for demographic anisotropy (e.g., age, gender, BMI). Future holistic risk assessments must inevitably incorporate the physiological impact of cognitive overload and operator stress.

Regarding the normative framework, the publication of ISO 10218-2:2025 represents the current technological standard, integrating numerous prior recommendations into strictly binding procedures. Although the preparatory phases for the revision of ISO/TS 15066 are currently underway, the release of radically new regulations cannot be predicted in the short term. Consequently, future standardization efforts must primarily address the normative acceptance of virtual validation. Furthermore, they must establish exact, hardware-agnostic protocols for measuring the total latency of braking chains. Overcoming these metrological and normative deficits constitutes the final barrier to the full-scale industrial implementation of intelligent collaborative workspaces.

## Figures and Tables

**Table 1:** Comparative synthesis of selected normative modifications between ISO 10218:2011 [13,14] and ISO 10218:2025 [9,15].

Evaluation Parameter	ISO 10218:2011 & ISO/TS 15066 [10,13,14]	ISO 10218:2025 [9,15]
Cybersecurity & IT/OT	Unspecified within the normative framework.	Mandatory risk assessment; enforcement of checksums and physical/software port deactivation.
Hazard Identification	Heavy reliance on normative hazard checklists.	Checklists reduced to informative annexes; safety evaluation driven exclusively by explicit Risk Assessment methodology (ISO 12100:2010).
Scope & Exclusions	Focused primarily on stationary industrial manipulators.	Explicit inclusion of mobile robotics terminology; strictly defined list of out-of-scope applications.
Technical Documentation	Fragmented documentation requirements.	Standardized documentation formats unified via interconnected normative references (explicit compliance with ISO 20607:2019).
Ergonomics & Psychology	Restricted to biomechanical and physical injury metrics.	Formal acknowledgement of psychological operator stress as a relevant residual risk factor.
Safety Verification	Physical measurement implied by subsequent technical specifications (ISO/TS 15066).	Strict mandate for physical measurement; mathematical estimations and simulations are explicitly insufficient for final validation.
Role of Simulation	Unaddressed in the context of safety validation.	Marginally acknowledged exclusively for the spatial detection of potential measurement points.

**Table 2:** Synthesis of the current state-of-the-art and future research trajectories regarding the safety evaluation of collaborative Power and Force Limiting (PFL) applications.

Domain	State-of-the-Art	Research Trajectories
Sensor technology, metrology, and signal processing	Commercial 2D foils and FSR matrices empirically exhibit limit saturation (clipping), local bulging, measuring system oscillations, and nonlinear signal filtering. They demonstrate high data dispersion, while a rigorous application of GUM uncertainty models for millisecond transient impacts is completely absent.	Analytical formulation of exact mathematical models representing the cascaded propagation of dynamic errors. Development and standardization of robust inverse deconvolution algorithms to reconstruct the actual contact force profile from distorted sensory data.
Material architecture and biofidelity	Conventional measurement devices physically and electronically fail. Although developing 3D conformal structures and e-skin prototypes achieve defined biofidelity under laboratory conditions (primarily for 1D normal forces), valid empirical data regarding their viscoelastic fatigue and long-term structural stability remain absent.	Construction of 3D multiaxial sensors for the continuous mapping of tangential and shear stresses. Empirical MTBF (Mean Time Between Failures) testing regarding the degradation and long-term mechanical durability of compliant materials under industrial loads. Development of “worst-case” biofidelic materials covering demographic extremes.
Boundary conditions and spatial-structural validation	Extreme testing polarization and physical discrepancies exist between quasi-static clamping and unconstrained transient impacts. Test apparatuses (PFMDs) operate in isolation as “black-box” systems, exhibiting an enormous reduction of the problem to 1D normal forces, utilizing highly simplified mathematical extrapolation factors for unconstrained collisions.	Implementation of real-time mechatronic data fusion (integrating the manipulator’s effective mass into the diagnostic loop). Formulation of dedicated models for sequential transient impacts (“push-to-clamp” scenarios) and the continuous mapping of friction coefficients determining the onset of actual lacerations.
Virtual validation, algorithmization, and software latency	The current normative framework (ISO 10218-2) blocks the integration of innovative approaches by rigidly fixating metrics strictly on surface pressure and normal force. The transition toward digital twins encounters the severe heterogeneity of computational solvers. Internal software collision detection generates latency that lacks independent and standardized quantification.	Normative unification of differential equations and coefficients (viscous damping, inertial mass distribution). Definitive specification of hardware-agnostic testing protocols for the objective measurement of total braking chain latency (including internal detection algorithms). Normative acceptance of predictive computational models.
Proactive mitigation, data fusion, and the human factor	Reactive mitigation exhibits a rigid polarization between PFL and SSM testing regimes. Localized applications of proximity sensors suffer from severe communication latency. Biomechanical limits remain fixed, broadly generalized, and deterministic; they completely abstract from physiological fluctuations, cognitive fatigue, and stochastic trajectory deviations of the operator.	Mathematical-software fusion of the PFL and SSM regimes, implementing AI, RGB-D, and AR/VR/MR for predictive evasive maneuvers. Dynamic scaling and stratification of safety models based on psychophysiological stress and demographic covariates through the integration of multimodal sensors (e.g., EMG) into event-driven full-body e-skins.

## Data Availability

No new data were created or analysed in this study. Data sharing is not applicable to this article.

## Appendix A. Systematic Overview and Technical Synthesis of the Analysed Literature

The presented appendix summarizes the final sample of 68 publications that successfully passed the rigorous screening process, constituting the foundational dataset of this study. The tabular arrangement is designed for maximum clarity, enabling rapid orientation within the specific technical parameters of the individual studies. Each entry details the respective authors, temporal classification, and publication medium. The abbreviation J (Journal) designates an article published within a peer-reviewed scientific journal, whereas the abbreviation C (Conference) denotes a paper published within scientific conference proceedings.

The analytical core of the Table A1 comprises a concise technical synthesis. This synthesis specifically deconstructs the methodological contributions and explicitly defines the physical or metrological limits of the given publication. The concluding section features exact cross-reference mapping to the defined Research Questions (RQ1–RQ4) and technological categories (C1–C5), indicated by the “•” symbol. To preserve the explicit continuity of longitudinal development within the domain of pHRI metrology, all records are ordered strictly chronologically, from the oldest to the most recent.

**Table A1:** Systematic overview and categorization of the publications constituting the final dataset for evaluating the metrological parameters of PFL applications.

Author	Year	Type	Description	RQ1	RQ2	RQ3	RQ4	C1	C2	C3	C4	C5
Povse et al. [65]	2012	C	This study describes the development of a mechatronic human arm substitute (PMLA) utilizing prosthetic materials to emulate the biomechanical response during robotic collisions. Experimental validation confirmed the device’s capability to predict tissue traumatization by measuring impact forces and energy density with a high degree of biofidelity. However, the results are strictly limited by low contact forces and simplified impactor geometries. This deficit severely complicates the direct application of the acquired data to high-speed industrial incidents.		•		•	•				•
Matthias et al. [44]	2014	C	This paper presents an experimental test stand featuring a linear guide and an adjustable spring stiffness mechanism. It is designed to emulate the mechanical compliance of various human body segments during pHRI collisions. The apparatus enables a comparative analysis between unconstrained free impacts and constrained clamping states utilizing a six-axis force/torque sensor and pressure sensory foils for mapping the spatial pressure distribution. However, the methodology exhibits a severe dependence on the problematic metrological reliability of pressure foils during transient events. The authors attempt to correct this physical deficit solely through software calibration rather than rigorous hardware optimization.	•	•	•	•	•	•			
Cordero et al. [35]	2014	J	This publication proposes and experimentally verifies the NIR (New Index for Robots) safety metric through simulations of manipulator collisions with a human head. Consequently, it adapts established traumatological metrics from the automotive industry for early-stage engineering risk assessment within collaborative robotics.	•	•		•		•	•		
Kim et al. [45]	2015	C	This study analyses the development of a 3D-printed soft robotic skin featuring a hermetic cavity. This structure simultaneously functions as a pneumatic damper for impact energy dissipation and as a sensor for regulating contact forces. Metrological validation proved a significant reduction in force amplitudes and a prolongation of the collision event, which the authors demonstrated during the manipulation of fragile objects. Nevertheless, the experimental apparatus operates strictly within sub-normative energy spectra utilizing minimal inertial masses. This metrological deficit renders the acquired data practically unusable for the objective certification of industrial PFL applications according to the ISO 10218-2 standard.	•		•		•			•	
Povse et al. [53]	2015	J	This work presents the design and validation of a passive mechanical arm (PMLA). It serves as a biofidelic dummy sensor for detecting contusions and lacerations during robotic collisions. The mechanical architecture derives from an analytical model of the human limb. Its parameters were optimized using comparative experiments with data acquired from direct impacts on a human volunteer. However, the primary limitation lies in the passive nature of the system. It remains fundamentally incapable of simulating the myotatic reflex and active muscle contraction. This absence alters the dynamic impedance of the body, leading to an inaccurate prediction of kinetic energy transfer during an actual impact. Furthermore, validation executed on a single subject physically prevents the statistical generalization of viscoelastic properties for the broader population.	•	•	•	•	•		•		•
Dagalakis et al. [37]	2016	J	The authors developed specialized measurement instrumentation designed for dynamic impact testing and the physical calibration of biofidelic artifacts. This apparatus serves the experimental verification of the mechanical durability of artificial tissues during direct contact with robotic end-effectors.	•	•	•	•	•	•			
Wang et al. [55]	2017	C	This paper integrates theoretical risk analysis with the experimental testing of static and dynamic collisions. The primary objective is to define the specific limit thresholds for mechanical loading and to formalize strict evaluation protocols ensuring the functional safety of collaborative robotic workspaces.		•		•		•			
Rosenstrauch & Krüger [76]	2017	C	This study subjects the ISO/TS 15066 specification to critical analysis through the experimental modeling of quasi-static contact between a robotic gripper and a biofidelic tissue simulant. The results definitively prove that the normative force limits are physically inadequate for preventing tissue traumatization. Specifically, tissue penetration and the onset of lacerations occur at values substantially below the established 140 N threshold. The study explicitly highlights the severe risk associated with shear stresses. Furthermore, it concludes that contemporary safety standards are improperly derived from subjective pain perception rather than the actual structural mechanical resistance of the human body.		•		•		•			•
Dombrowski et al. [78]	2018	C	This study presents the development of a simulation module for the Process Simulate environment. This module enables the virtual prediction of contact forces and pressures within PFL applications directly during the digital planning phase. Based on a parametric analysis of real physical measurements, the authors identified the deterministic influence of effective mass, velocity, and impactor geometry on the resulting mechanical loading of the tissue. The proposed numerical solution approximates both transient and quasi-static phenomena. Consequently, it eliminates the necessity for time-consuming physical testing during the commissioning phase of the workspace. Despite the high utility for virtual validation, the authors explicitly highlight the necessity for further refinement of the mathematical models to achieve full metrological reliability.		•		•		•	•		
Schlotzhauer et al. [72]	2019	C	This research analyses biomechanical loading during collisions through the spatial mapping and construction of a statistical model (2D Collision-force-map). Based on the specific configuration of the robotic arms and the reflected mass, this model predicts transient forces across the entire operational workspace. Consequently, it proves the physical inadequacy of isolated point-based safety evaluations. This approach enables the optimization of trajectories without the need for continuous experimental physical testing.		•		•			•		
Shin et al. [62]	2019	J	This paper presents an analytical methodology for approximating contact pressure in real-time. It utilizes a modified Hertzian contact theory combined with a hyperelastic Mooney–Rivlin model. The proposed mathematical apparatus replaces computationally demanding FEM simulations, enabling load prediction directly within the robot’s control system. This methodology was validated through experiments utilizing silicone elastomers. However, the model fundamentally ignores the viscoelastic energy dissipation typical of living biological tissue. Furthermore, it relies exclusively on the theoretical derivation of pressure from axial force, lacking direct metrological verification of the spatial stress distribution.		•		•			•	•	
Park et al. [84]	2019	J	This publication presents an in vivo study executed on 100 human volunteers. It focuses on quantifying Pressure Pain Thresholds (PPT) across 29 distinct anatomical locations utilizing a manual algometer to validate the limits defined in the ISO/TS 15066 norm. The results demonstrate a significant statistical correlation between the pain threshold, gender, and the surface area of the impactor. The measured values are generally lower than current normative standards. However, the methodology exhibits a severe metrological deficit due to the near-total absence of dynamic loading. The quasi-static measurements fundamentally ignore the strain-rate dependent, nonlinear viscoelastic behaviour of actual tissue. Consequently, this data remains metrologically inadequate for evaluating high-speed transient impacts within industrial robotics.				•					•
Iki et al. [40]	2020	C	This research implements viscoelastic parameters, extracted directly from experimental measurements on human subjects, into the construction of a proprietary biofidelic sensor (dummy skin) mounted on a linear guide. This mechanical architecture ensures the exact physical replication of the nonlinear mechanical properties of the human hand. It serves the rigorous validation of collision forces in strict accordance with the ISO/TS 15066 specification.	•	•	•	•	•				•
Ganglbauer et al. [64]	2020	C	This research presents software architecture designed for the dynamic prediction of safe velocity limits within the PFL regime. The algorithm fuses the visual detection of the human skeletal structure with the real-time calculation of instantaneous effective mass and the application of the Hertzian contact model. By executing an online approximation of the physical collision event, the system effectively eliminates the necessity for time-consuming experimental re-measurement across every kinematic configuration.		•		•			•	•	
Staab et al. [26]	2020	C	This research from the ABB corporate center presents an innovative inversion of the measurement chain. A biofidelic load cell is integrated directly onto the manipulator flange, actively striking a pendulum mechanism equipped with a variable reflected mass. This configuration is designed for the exact simulation of unconstrained free transient impacts. The methodology enables the rigorous validation of analytical models by calculating mechanical work directly from the pendulum’s angular deflection. However, it strictly requires advanced algorithmic compensation for inertial forces, as the mass of the sensor becomes an integral component of the impactor’s total effective mass. Therefore, the paper defines a highly specific testing procedure to increase the reproducibility of dynamic measurements, critically addressing the influence of dynamic errors introduced into the data during the spatial movement of the sensor.	•	•		•		•			
Heng et al. [77]	2020	J	This research implements an inverse safety methodology. Instead of utilizing an external biofidelic measurement device, a rigid sensor is deployed in combination with a viscoelastic damping layer integrated directly onto the physical structure of the robot. This structural modification serves to dynamically simulate the mechanical response of actual human tissue during a collision.		•			•			•	
Scibilia et al. [27]	2021	J	This critical interlaboratory analysis demonstrates significant statistical discrepancies in the data measured during PFL regime testing across different institutions. Through the systematic deconstruction of the utilized metrological chains, the authors identify non-standardized instrumentation and the subjective interpretation of normative requirements as the primary physical barriers preventing the achievement of objective and fully reproducible safety in pHRI.	•	•	•	•		•			
Valori et al. [20]	2021	J	This methodological study maps the legislative transition of measurement procedures from the ISO/TS 15066 technical specification into the newly revised ISO 10218-2 norm (specifically Annex N). It precisely defines standardized testing protocols for the validation of both transient and quasi-static collisions. Subsequently, the authors demonstrate these protocols through the experimental determination of safe operational velocities for a UR10 manipulator utilizing a certified PFMD.	•	•		•		•			
Rajaei et al. [36]	2021	C	This study experimentally quantifies the highly nonlinear deformational characteristics of soft human arm tissue during both transient and quasi-static impacts. Through the analysis of the acquired force-deformation curves, the authors define the fundamental biomechanical parameters strictly required for the development of advanced and biofidelic measurement devices.	•	•	•	•			•		•
Case et al. [52]	2021	C	This study critically addresses the detrimental influence of the high local stiffness inherent to conventional diagnostic instruments on measurement accuracy. The authors propose the development of a soft capacitive sensor whose material architecture strictly replicates the viscoelastic properties of the human forearm. The engineering solution exploits the linear dependence of electrical capacitance on applied force to exactly detect contact parameters. Consequently, it creates a biofidelic technological alternative to highly rigid measurement systems. By directly integrating the biomechanical characterization of human tissue into the sensor’s physical construction, this work defines a new metrological standard for the future development of dummy arms intended for pHRI safety validation.	•		•	•	•				•
Kirschner et al. [74]	2021	C	This study critically reflects upon the absence of rigorous metrological standards for evaluating the force perception and reaction time of robotic manipulators. The authors propose a comprehensive reference framework for the objective benchmarking of tactile performance. Utilizing specific measurement protocols and hardware configurations, the research effectively isolates force control capabilities. The authors demonstrate this methodology through a direct comparison of the UR10e, UR5e, and Franka Emika Panda platforms. Although the metrological purity of the results may be compromised by internal signal filtering within the proprietary closed controllers, the work provides a unified methodological tool for the objective assessment of a manipulator’s technical capability for dynamic pHRI scenarios. Thus, it supplements the missing normative metrics alongside existing standards governing purely kinematic precision.		•		•		•			
Švarný et al. [43]	2021	J	This work expands spatial safety evaluation into the 3D operational workspace of the manipulator by generating nonlinear collision maps. Based on an experimentally verified mathematical model, these maps predict maximum impact forces dependent on the specific joint configuration. Consequently, this methodology enables the exact identification of safe zones directly during trajectory planning, effectively eliminating the need for comprehensive spatial physical testing.	•	•		•			•		
Kirschner et al. [63]	2021	C	This study critically deconstructs the simplified spring model mandated by the ISO/TS 15066 technical specification. Based on an empirical analysis of UR10e, UR5e, and Franka Emika Panda manipulators, the authors prove its physical inability to accurately predict actual clamping forces. This failure stems from the model’s fundamental ignorance of reflected inertia and the latency of internal safety algorithms. The authors introduce the methodological concept of Constrained Collision Force Maps (CCFM). These maps systematically correlate impact velocity, detection thresholds, and contact stiffness with force amplitude. This methodology provides an exact engineering tool for parameterizing safe workspaces, successfully overcoming the severe metrological limits of the current normative methodology.		•		•		•			
Herbster et al. [60]	2021	C	This research investigates the mathematical transformation of data acquired by rigid sensors into values corresponding precisely to the dynamics of unconstrained free collisions with the human body. This approach effectively eliminates the conservative overestimation of force peaks during non-clamping scenarios. Consequently, it enables the optimization of safe operational velocity limits for collaborative robots and highlights the need for specialized compliant hardware.		•		•		•	•		
Lachner et al. [56]	2021	J	This research proposes the implementation of an impedance control algorithm based on energy budgets. This algorithm dynamically restricts the mechanical energy of the robot in real-time, regardless of its instantaneous kinematic configuration. Thus, it ensures inherent compliance with the ISO/TS 15066 safety limits strictly through software control, entirely eliminating the reliance on external hardware sensor instrumentation.		•		•				•	
Kirschner et al. [46]	2021	C	This paper introduces the concept of a Contact Sensitivity Map (CSM) designed for the objective benchmarking of internal collision detection systems across various kinematic configurations in collaborative robots. The protocol visually maps the minimal external stimuli required to trigger a safety response. The results demonstrate severe anisotropy, proving that detection sensitivity remains highly dependent upon the manipulator’s exact position within the workspace. However, the methodology remains critically dependent on the accuracy of internal dynamic models and rigorous friction compensation. Furthermore, it fundamentally ignores the impact of communication bus latency on detection reliability at higher velocities. Therefore, while the proposed approach offers a comparative framework for robotic proprioceptive capabilities, it simultaneously introduces metrological uncertainty strictly tied to the hardware limits of proprietary control systems.	•		•	•		•	•		
Hirata et al. [39]	2021	C	This study presents the development of an anthropomorphic testing device featuring a multilayer structure composed of silicone and urethane gels. This material architecture exactly emulates the mechanical properties of human skin, fat, and bone for pain detection during pHRI. Integrated arrays of pressure sensors enable the high-resolution measurement of stress distribution within the tissues. The biofidelity of the prototype was validated through direct comparison with in vivo data acquired during both quasi-static and dynamic impacts. Despite the sophisticated nonlinear tissue model, the practical utility of the study remains severely limited by the low testing velocities employed (0.7 m/s). These low velocities reflect neither the actual dynamics of high-speed industrial collisions nor the dynamic frequency response of the utilized sensors.	•	•	•	•	•				•
Schneider et al. [31]	2021	C	This study empirically quantifies the influence of supplementary damping and the geometry of collision surfaces on the active reduction in transient forces during impacts directed at the human shoulder region. By comparing actual measured data with the analytical models derived from ISO/TS 15066, the authors prove that modifying the robot’s exterior hardware surface enables a measurable increase in operational velocity while strictly adhering to biomechanical limits. The resulting empirical database serves as an exact foundation for rigorous risk assessment processes and the verification of theoretical calculations within real industrial contexts.	•	•		•				•	
Schlotzhauer et al. [33]	2022	C	This study presents an automated testing environment designed for the systematic generation of collision data. This data serves to saturate an expert system explicitly intended for the virtual validation of workspaces operating within the PFL regime. The core of the proposed architecture is a specific module dedicated to multidimensional data approximation and load estimation. This module enables the prediction of biomechanical loading during the pre-integration phase. Furthermore, it autonomously generates precise proposals for adjusting process parameters (specifically velocity and payload mass). This predictive methodology effectively eliminates the economic risks inherently associated with reactive safety assurance approaches.	•	•		•		•	•		
Fischer et al. [30]	2022	C	This study critically analyses the absence of a standardized methodology for the mechanical mounting of diagnostic devices (PFMD) during the measurement of unconstrained transient contacts. Through a rigorous comparative analysis of three distinct configurations—rigid fixation, a sliding linear guide, and a pendulum mount—the authors quantify the deterministic influence of these boundary conditions on the acquired data. Based on analytical models and physical experiments, the research formulates exact engineering recommendations specifically designed to eliminate this metrological vacuum and ensure the biofidelic simulation of dynamic events within real collaborative systems.	•	•		•		•			
Švarný et al. [75]	2022	J	Based on an extensive dataset comprising 2250 measurements, this study rigorously evaluates the impact of commercial sensory skin on the mitigation of collision forces for both collaborative and standard industrial manipulators. The research explicitly separates the passive damping effect of the polymeric material from the active sensory response of the system to ensure strict compliance with PFL regime requirements.	•	•		•				•	
Zimmermann et al. [7]	2022	J	This study subjects commercial PFMDs to a rigorous comparative analysis, exposing critical inter-instrumental variability during the detection of physically identical transient impacts. The research exactly proves that the differing material architectures of the damping elements (elastomers and springs) utilized by individual manufacturers directly generate significant deviations in the measured forces and pressures. This metrological discrepancy, stemming from vague technical specifications within the governing standards, severely degrades the objectivity and comparability of industrial safety audits. The paper thus isolates the hardware error rate of the instruments as the primary source of metrological inconsistency, critically questioning the fundamental validity of contemporary certification processes executed within the PFL regime.	•	•	•	•		•			
Nguyen a Case [81]	2022	J	This research identifies a severe hardware deficit regarding the sensory drift of current sensors induced by temperature fluctuations and physical degradation. This drift negatively affects the accuracy of proprioceptive collision detection. As a technological solution, the authors propose a compensation algorithm based on an Artificial Neural Network (ANN). This algorithm continuously models these thermal and degradational fluctuations, thereby refining the internal estimation of contact forces. This ensures the highly reliable execution of the PFL safety function without necessitating any external sensory instrumentation.				•				•	
Huang et al. [73]	2022	J	This study critically addresses the severely limited bandwidth of standard Generalized Momentum Observers (GMO), a deficit that causes unacceptable temporal latency during the detection of high-frequency transient collisions. By implementing a specifically modified Back-Input Compensation algorithm, the authors successfully eliminated systemic model deviations and signal noise. This modification enables the highly sensitive, real-time detection of both hard and soft impacts. Validation employing external biofidelic instrumentation definitively proved that this software optimization of the control loop actively reduces dissipated kinetic energy by ensuring the timely initiation of the braking trajectory. Consequently, it guarantees strict compliance with safety limits without necessitating any physical hardware modifications to the manipulator.		•		•				•	
Byner et al. [57]	2022	C	This study subjects the standard analytical models defined in ISO/TS 15066 to critical scrutiny due to their fundamental ignorance of the machine’s active dynamic reaction. The authors propose an expanded two-mass model that exactly integrates the latency of internal collision detection and the manipulator’s specific braking profile directly into the prediction of contact forces during clamping scenarios. This mathematical apparatus, validated empirically on an ABB CRB15000 robot, serves as a robust predictive tool for risk assessment. It successfully overcomes the disproportionate conservatism of existing normative standards and enables the exact software optimization of safe operational velocities without the necessity for iterative physical testing.		•		•			•		
Steinecker et al. [71]	2022	C	This paper analytically defines the concept of Mean Reflected Mass (MRM) as a deterministic metric for the optimization of safe trajectories in collaborative robotics. The authors rigorously account for the anisotropic nature of effective mass by mathematically integrating its specific values across all directional vectors. This approach eliminates the physical unpredictability of the exact impact vector. For redundant manipulators, this methodology exploits the kinematic null space to calculate a posture that actively minimizes inertial effects during a potential collision, critically without compromising the execution of the primary operational task.		•		•			•	•	
Clever et al. [51]	2022	C	This study criticizes the contemporary safety evaluation methodology defined by ISO/TS 15066. The authors propose replacing the metrologically problematic measurement of peak forces and pressures with mathematically robust integral quantities, specifically energy transfer and power flux density. Experiments executed utilizing an ABB GoFa robot and a CBSF sensor confirm that this approach more effectively eliminates high-frequency signal noise. However, its exact accuracy remains critically dependent upon the flawless temporal synchronization of the acquired data streams. Furthermore, practical industrial application is severely limited by the reliance on idealized geometric models for calculating the contact area. Without direct dynamic spatial measurement, this theoretical approximation directly leads to the conservative overestimation of the actual collision parameters.	•	•	•	•		•			
Mujica et al. [87]	2022	C	This study models the physical co-manipulation of payloads between a human operator and a KUKA LBR iiwa robot within the Matlab environment. The simulation exactly integrates an impedance model of the human arm based entirely on the equilibrium point hypothesis. Subsequent physical validation, utilizing the actual manipulator operating in admittance mode, confirmed a high correlation between simulated and real interaction forces during smooth, continuous motion. However, the proposed computational framework remains entirely unusable for the rigorous safety validation of sudden collisions. The model fundamentally ignores high-frequency transient impact events and the highly nonlinear dissipation of kinetic energy within soft biological tissues. This severe structural limitation restricts its application solely to predicting forces during low-speed, cooperative co-manipulation.				•			•		
St-Jean et al. [67]	2023	J	This publication analyses the mechanical integration of magnetorheological actuators directly into the structural design of collaborative arms to actively reduce effective inertia. Through rigorous experimental testing, the authors demonstrate a significant reduction in transient impact forces compared to conventional joint drives. This physical hardware modification is executed simultaneously with the implementation of advanced collision detection algorithms based directly on the mathematical filtration of angular velocity.		•		•				•	
Jeanneau et al. [83]	2023	J	This work focuses on the mathematical reduction of the dynamic model of a collaborative manipulator to a simplified mass-spring-mass translational system. Utilizing nonlinear Hertzian contact theory, this model predicts impact forces. Consequently, it serves to optimize the mechatronic design of the robot to achieve the dynamic decoupling of inertial masses.				•			•		
Herbster et al. [49]	2023	J	This study presents a macroscopic, unidimensional Simulink model designed for calculating contact forces during clamping scenarios. The model mathematically approximates the dynamic response of both the measurement device and human tissue. The primary objective is to replace economically demanding physical experiments with a predictive computational tool for determining safe manipulator velocities.	•	•		•		•	•		
Hüsing et al. [34]	2023	C	This research critically examines the near-complete absence of exact specifications regarding the edge geometry of end-effectors within safety standards (specifically ISO/TS 15066). Through an extensive experimental campaign, the authors quantify the deterministic influence of edge rounding and chamfering on the reduction in contact pressure during quasi-static collisions. Although the study saturates a critical data vacuum concerning hardware-based risk prevention, its metrological validity remains severely limited. This limitation stems from the fundamental dependence of the exact contact area upon the highly nonlinear mechanical impedance of the utilized sensor. Furthermore, the risk of pressure clipping (measurement element saturation) compromises the results. Without a standardized viscoelastic characterization of the testing interface, the transferability of these specific findings to actual living human tissue is fundamentally restricted.	•	•		•				•	
Zurlo et al. [86]	2023	C	This paper presents software architecture designed for the sensor-less detection and spatial localization of the exact contact point. The algorithm fuses energy monitoring and generalized momentum observer methodologies to estimate collision forces directly from the internal state data of the joint motors. Subsequently, it applies an advanced particle filter to achieve the precise spatial isolation of the impact location.				•				•	
Kovinčić et al. [58]	2023	C	This paper critically reflects upon the inherent inflexibility of the normative validation methods defined by ISO/TS 15066. The authors propose a hybrid predictive computational model based directly on physics-informed machine learning. The algorithm successfully fuses the linearized kinematics of the manipulator with an ensemble of decision trees and a dedicated neural network. Utilizing training data acquired from a UR10e robot, this methodology enables the exact mathematical estimation of maximum safe operational velocities. Consequently, it eliminates the strict necessity for redundant physical measurements whenever the workspace configuration is modified.		•		•			•		
Schneider et al. [32]	2023	C	This conference study defines a specific methodology for the empirical modeling of maximum permissible velocities within the PFL regime, utilizing a CNC machine tending application as a primary example. The research exactly quantifies the physical influence of payload mass, manipulator pose, and supplementary damping layers on the overall level of dissipated kinetic energy. The primary engineering output is a mathematical approximation equation specifically formulated to predict limit velocities for quasi-static events. This equation serves as a surrogate computational model, successfully replacing economically demanding and iterative physical testing. It provides a robust predictive apparatus for efficiently executing risk analysis during ongoing modifications to the workspace configuration.	•	•		•		•		•	
Ponikelský et al. [68]	2023	J	This application study presents the strict experimental validation of an actual industrial palletizing workspace operating a UR10e manipulator. Through rigorous biofidelic measurement, the audit detected critical violations of the biomechanical limits defined by ISO/TS 15066 (with force peaks reaching up to 452.9 N). This work serves as a detailed engineering risk evaluation. Based exclusively on the measured data, it definitively proves the non-compliant status of the specific workspace for PFL operation. Consequently, it proposes the necessity of reconfiguring the safety chain by integrating laser scanners to enforce a Speed and Separation Monitoring (SSM) regime.		•		•		•			
Fischer et al. [41]	2023	J	This study systematically quantifies the severe influence of impactor geometry and boundary fixation conditions on the measured values of transient forces and pressures. Through exact physical experimentation, the authors prove that neglecting these specific parameters within normative testing procedures directly generates high metrological error rates. This physical omission fundamentally prevents the objective validation of safety limits as dictated by ISO/TS 15066.	•	•	•	•		•			
Liu et al. [42]	2023	J	This study critically reflects upon the severe mechanical limits of conventional measurement instruments based strictly on linear springs. The authors present a comprehensive mechatronic design for a highly biofidelic testing dummy. The hardware structure of this prototype directly materializes the exact nonlinear viscoelastic properties of human tissue. The engineering core of this work is the direct physical transformation of empirically acquired mechanical impedance parameters (derived from human subjects) into a functional sensor prototype. This instrument strictly replicates the specific dynamics of energy dissipation occurring during biological deformation. Consequently, the resulting metrological instrument successfully eliminates the systematic errors plaguing simplified commercial devices. It provides a scientifically robust apparatus for the objective validation of both transient and quasi-static collisions within unstructured HRC environments.	•	•	•	•	•		•		•
Samarathunga et al. [24]	2024	J	This paper critically analyses the dynamic mechanical characteristics of a commercial PFMD sensor utilizing a simple single-degree-of-freedom system model. Through physical experimentation, the authors prove its completely inadequate frequency response and severely low structural damping during the detection of transient impact events. This exact metrological failure directly leads to an urgent requirement for the rigorous normative specification of the dynamic physical parameters governing these measurement devices.	•	•		•		•	•		
Samarathunga et al. [54]	2024	C	This work utilizes a pendulum mechanism to specifically measure transient impacts. This approach effectively replaces complex biofidelic sensors by relying on the direct physical analysis of force transfer onto a freely oscillating mass. The authors critically deconstruct the theoretical calculations defined in ISO/TS 15066 and introduce an analytical computational model for determining the robot’s exact effective mass utilizing the Jacobian matrix. However, empirical validation remains strictly limited by the applied low testing velocity of $700\;\frac{mm}{s}$ and the utilization of a completely rigid impactor. This experimental methodology fundamentally omits the highly nonlinear viscoelastic deformation of living tissue, a characteristic that remains completely typical for actual unstructured industrial collisions.	•	•		•		•	•		
Zhu et al. [25]	2024	J	This methodological study fundamentally deconstructs the entire metrological measurement chain as defined by the ISO/TS 15066 standard. The authors explicitly identify the primary structural sources of metrological deviations occurring within pHRI applications. To significantly increase overall data consistency, the authors propose replacing the direct physical measurement of transient forces with a robust computational model. This proposed methodology utilizes a highly precise laser tracker combined with a mathematically modified approximation of the manipulator’s effective mass.	•	•		•		•		•	
Li [80]	2024	C	This publication presents the development of a biofidelic artificial finger featuring an integrated PVDF sensor. This specific sensor architecture is capable of continuously detecting both shear deformations and high-frequency vibrations during highly complex contact scenarios (e.g., pressure combined with mechanical slip). The study rigorously validates the mathematical correlation between internal deformation energy and the applied reference mechanical work. Concurrently, the authors explicitly identify and critically analyse the remaining technical deficits specifically concerning the exact spatial localization of the contact point.			•		•				
Hornung et al. [50]	2024	C	This research specifically focuses on the comprehensive development and rigorous validation of a Finite Element Method (FEM) numerical model representing a standard PFMD. Through an extensive parametric computational study explicitly analysing damping coefficients and specific material formulations, the authors simulate transient collisions with a human hand. The primary objective is to successfully replace economically demanding physical experimental testing with highly accurate, predictive computational methodologies.	•			•			•		
Ponikelský et al. [79]	2024	J	This research rigorously maps the highly asymmetrical operational workspace of the UR10 manipulator. The study definitively proves a severe escalation in actual contact forces dynamically dependent upon the physical proximity to the robot’s static base. Furthermore, the authors explicitly identify a critical methodological error regarding the selection of an inadequate operational measurement range for the utilized pressure foils. Due to immediate physical saturation (clipping), these foils completely prevented the valid quantification of dynamic pressure peaks during higher-speed impact testing.			•	•		•			
Rustler et al. [47]	2024	C	This study rigorously evaluates the practical implementation of an adaptive electronic skin on a UR10e robot. The authors physically prove that the continuous, dynamic modulation of sensitivity threshold values—dependent strictly upon the instantaneous velocity and the dynamic effective mass of the individual robotic links—enables the absolute maximization of operational performance. This performance increase is achieved while maintaining strict, mathematically guaranteed compliance with the normative safety limits defined by ISO/TS 15066.	•	•	•	•	•				
Kovinčić et al. [59]	2024	J	This publication significantly expands upon the previous methodology presented in [the corresponding paper] by integrating a rigorous mathematical apparatus and advanced spatial mapping techniques. The authors utilize an exact impact model to systematically generate physical features specifically required for training a machine learning ensemble. By strictly applying advanced interpolation techniques to the resulting data distribution spectrum, the authors successfully extrapolate maximum safe impact velocities directly into the form of a continuous 3D spatial map. This comprehensive output serves as an exact engineering tool for the complete validation of the entire collaborative workspace.		•		•			•		
Han et al. [61]	2024	J	This study analyses dynamic pain thresholds during high-speed impacts utilizing a pendulum mechanism tested on 37 human subjects. The specific methodology rigorously compares the physical influence of a wedge impactor versus a cylindrical impactor. The empirical results definitively confirm that a sharper geometric profile systematically induces physical pain at significantly lower absolute forces due to a higher spatial stress concentration. The authors exactly quantified this finding utilizing a log-logistic model targeted at the 75th percentile of the testing population. However, the critical technical deficit of this study is the absolute metrological failure of the utilized foil pressure sensors. Due strictly to their severely inadequate sampling frequency and poor dynamic frequency response, the sensors completely failed to physically capture the transient pressure peaks. This total hardware failure resulted directly in the complete elimination of all acquired pressure data from the final statistical analysis.		•	•	•		•			•
Emiliani et al. [82]	2024	J	This work describes the structural design of a collaborative workspace utilizing a UR10e robot and custom mechatronic subsystems for assembling bearings and retaining rings. Operator safety is ensured by a laser scanner dynamically modulating the operational velocity, combined with topologically optimized SLA end-effector fingers. The functional validation of the system was executed through the rigorous metrological measurement of forces and pressures utilizing a certified GTE CoboSafe instrument. The acquired transient and quasi-static values were subsequently verified experimentally against the strict limits defined by the ISO/TS 15066 specification.				•		•		•	
D’Antona et al. [29]	2025	J	This study presents the development and validation of a Variable Stiffness Impact Testing Device (VSITD). The apparatus utilizes a specific mechanical module featuring an adjustable spring preload combined with a piezoresistive sensory matrix to simulate the biomechanical compliance of the human body. The authors critically identify physical limits regarding stiffness saturation exceeding the $60\;\frac{N}{mm}$ threshold. Consequently, they highlight the importance of advanced metrological calibration specifically intended for industrial applications.	•		•	•	•	•	•		
Samarathunga et al. [21]	2025	J	This systematic review critically evaluates contemporary pHRI modeling methodologies. It identifies a significant metrological deficit in the mathematical prediction of nonlinear viscoelastic tissue dynamics during high-speed transient events. Furthermore, the study explicitly highlights the lack of standardized measurement protocols and rigorously exposes the demographic non-inclusivity of current normative safety limits.	•			•		•	•		
Fischer et al. [85]	2025	C	This work critically reflects upon the methodological absence of anthropometric variability within the ISO/TS 15066 technical specification. Based on an empirical analysis of a 31-participant sample, the authors prove that spatial uncertainty regarding the exact impact location—driven entirely by the operator’s physical proportions—deterministically generates diametrically opposed contact force values. This extreme physical variance strictly requires the direct integration of personalized biometric data into the formal risk assessment process.				•		•			
Zimmermann et al. [28]	2025	C	This study critically compares five commercial biofidelic measurement devices (PFMDs) during the standardized testing of three distinct collaborative manipulators. Based on the experimentally proven, significant data dispersion regarding the measured maximum forces, the authors explicitly identify the severe metrological inconsistency of contemporary validation methods. Consequently, they propose strictly required modifications to ongoing normative standardization processes.	•			•		•			
Suita a Okawa [88]	2025	J	This work focuses on the development of passive viscoelastic damping layers applied directly to the manipulator’s exoskeleton. Through the rigorous dynamic mechanical analysis of specific urethane materials, the authors experimentally prove the physical capability to effectively reduce transient collision forces strictly below normative limits. This structural mitigation is successfully achieved even at high operational velocities, entirely eliminating the necessity for active external sensory instrumentation.				•				•	
Sun et al. [48]	2025	C	This work presents an integrated testing system engineered for the synchronous, multimodal measurement of collision parameters. The proposed architecture successfully combines a physical dummy, equipped with multidimensional force sensors and inertial measurement units, with a corresponding virtual environment simulated within the Gazebo engine. This hybrid setup serves the exact virtual and physical verification of strict compliance with the safety limits defined by the ISO/TS 15066 standard.	•	•	•	•	•	•	•		
Balletshofer et al. [70]	2025	C	This research critically highlights the metrological failure of conventional analytical methods in determining reflected mass. The authors propose an empirical methodology termed the “Collision Mass Map.” By executing the spatial mapping of effective mass strictly based on real physical measurements, this methodology enables the exact prediction of transient forces. Consequently, it allows for the rigorous optimization of manipulator operational velocities while continuously guaranteeing absolute adherence to normative safety limits.		•		•			•		
Kóczi a Sárosi [69]	2025	J	This research utilizes a specifically designed experimental test stand equipped with a pneumatic muscle actuator to exactly differentiate between quasi-static and transient collisions. The authors critically identify the severe physical limits of low-cost resistive sensors. Specifically, when subjected to impact forces exceeding 150 N, these sensors exhibit a complete loss of signal linearity. Consequently, the mathematical correlation with the certified reference load cell completely collapses due to uncompensated dynamic phenomena.		•	•		•			•	
Palmieri et al. [66]	2025	C	This work executes a rigorous dynamic characterization of the commercial GTE CoboSafe sensor by mathematically approximating its mechanical architecture as a second-order system. Through the strict analysis of transient events induced by an impact hammer, the authors exactly identified the fundamental physical parameters, specifically its natural frequency, damping ratio, and effective mass. Utilizing reverse engineering methodologies, this research exposes the internal dynamic properties of an established measurement system. Consequently, it substantially supplements existing knowledge regarding the severe metrological variability plaguing instruments designed for collaborative safety validation.	•	•		•		•			
Caneschi et al. [38]	2026	J	This work focuses on the engineering development of a low-cost biofidelic prototype explicitly designed for collision validation strictly adhering to the ISO/PAS 5672:2023 specification. Subsequently, the research executes a rigorous comparative analysis between conventional analytical contact models and advanced machine learning algorithms. The authors explicitly identify decision tree ensembles as a significantly more precise computational method for predicting force effects within actual Power and Force Limiting (PFL) applications.	•	•		•	•	•	•		

## Appendix B. Quality Assessment and Evidence Weighting Table

This appendix details the quantitative evaluation of the 68 publications included in the final dataset, strictly applying the Quality Assessment (QA) methodology defined in Section 2.3. The objective of this table is to establish a rigorous bibliometric and methodological hierarchy, preventing the analytical equivalence of outdated or preliminary studies with contemporary, high-impact empirical research.

Each publication is evaluated across three primary dimensions (see Table A2): Source Rigor (SR), reflecting the stringency of the peer-review process; Methodological Rigor (MR), quantifying the exactness of the experimental boundary conditions and statistical evaluation; and the Temporal Relevance Index (TRI), which applies a temporal filter to identify obsolete hardware deficits. The algebraic sum of these components yields the Total Quality Score (TQS), generating the final classification into three distinct evidence tiers: High Confidence (8–9), Moderate Confidence (5–7), and Low/Historical Relevance (3–4).

**Table A2:** Complete Quality Assessment table calculating the Total Quality Score (TQS) and the resulting Evidence Level for each evaluated publication.

Author	Year	SR Score	MR Score	TRI Score	TQS	Evidence Level
Povse et al. [65]	2012	1	2	1	4	Low/Historical Relevance
Matthias et al. [44]	2014	1	2	1	4	Low/Historical Relevance
Cordero et al. [35]	2014	3	2	1	6	Moderate Confidence
Kim et al. [45]	2015	1	2	1	4	Low/Historical Relevance
Povse et al. [53]	2015	2	3	1	6	Moderate Confidence
Dagalakis et al. [37]	2016	2	2	1	5	Moderate Confidence
Wang et al. [55]	2017	1	1	2	4	Low/Historical Relevance
Rosenstrauch & Krüger [76]	2017	1	2	2	5	Moderate Confidence
Dombrowski et al. [78]	2018	1	1	2	4	Low/Historical Relevance
Schlotzhauer et al. [72]	2019	1	3	2	6	Moderate Confidence
Shin et al. [62]	2019	1	2	2	5	Moderate Confidence
Park et al. [84]	2019	3	3	2	8	High Confidence
Iki et al. [40]	2020	1	3	2	6	Moderate Confidence
Ganglbauer et al. [64]	2020	1	2	2	5	Moderate Confidence
Staab et al. [26]	2020	1	2	2	5	Moderate Confidence
Heng et al. [77]	2020	3	3	2	8	High Confidence
Scibilia et al. [27]	2021	3	3	2	8	High Confidence
Valori et al. [20]	2021	3	2	2	7	Moderate Confidence
Rajaei et al. [36]	2021	1	2	2	5	Moderate Confidence
Case et al. [52]	2021	1	2	2	5	Moderate Confidence
Kirschner et al. [74]	2021	1	3	2	6	Moderate Confidence
Švarný et al. [43]	2021	1	2	2	5	Moderate Confidence
Kirschner et al. [63]	2021	1	3	2	6	Moderate Confidence
Herbster et al. [60]	2021	1	2	2	5	Moderate Confidence
Lachner et al. [56]	2021	3	3	2	8	High Confidence
Kirschner et al. [46]	2021	1	2	2	5	Moderate Confidence
Hirata et al. [39]	2021	1	2	2	5	Moderate Confidence
Schneider et al. [31]	2021	1	2	2	5	Moderate Confidence
Schlotzhauer et al. [33]	2022	1	2	2	5	Moderate Confidence
Fischer et al. [30]	2022	1	3	2	6	Moderate Confidence
Švarný et al. [75]	2022	3	3	2	8	High Confidence
Zimmermann et al. [7]	2022	3	3	2	8	High Confidence
Nguyen a Case [81]	2022	3	3	2	8	High Confidence
Huang et al. [73]	2022	3	2	2	7	Moderate Confidence
Byner et al. [57]	2022	1	2	2	5	Moderate Confidence
Steinecker et al. [71]	2022	1	2	2	5	Moderate Confidence
Clever et al. [51]	2022	1	2	2	5	Moderate Confidence
Mujica et al. [87]	2023	1	2	2	5	Moderate Confidence
St-Jean et al. [67]	2023	3	3	3	9	High Confidence
Jeanneau et al. [83]	2023	3	3	3	9	High Confidence
Herbster et al. [49]	2023	3	2	3	8	High Confidence
Hüsing et al. [34]	2023	1	2	3	6	Moderate Confidence
Zurlo et al. [86]	2023	1	2	3	6	Moderate Confidence
Kovinčić et al. [58]	2023	1	2	3	6	Moderate Confidence
Schneider et al. [32]	2023	1	2	3	6	Moderate Confidence
Ponikelský et al. [68]	2023	2	2	3	7	Moderate Confidence
Fischer et al. [41]	2023	3	3	3	9	High Confidence
Liu et al. [42]	2023	3	2	3	8	High Confidence
Samarathunga et al. [24]	2024	3	3	3	9	High Confidence
Samarathunga et al. [54]	2024	1	2	3	6	Moderate Confidence
Zhu et al. [25]	2024	2	2	3	7	Moderate Confidence
Li [80]	2024	1	2	3	6	Moderate Confidence
Hornung et al. [50]	2024	1	3	3	7	Moderate Confidence
Ponikelský et al. [79]	2024	3	2	3	8	High Confidence
Rustler et al. [47]	2024	1	2	3	6	Moderate Confidence
Kovinčić et al. [59]	2024	3	2	3	8	High Confidence
Han et al. [61]	2024	3	3	3	9	High Confidence
Emiliani et al. [82]	2024	3	2	3	8	High Confidence
D’Antona et al. [29]	2025	3	3	3	9	High Confidence
Samarathunga et al. [21]	2025	3	3	3	9	High Confidence
Fischer et al. [85]	2025	1	3	3	7	Moderate Confidence
Zimmermann et al. [28]	2025	1	3	3	7	Moderate Confidence
Suita a Okawa [88]	2025	1	3	3	7	Moderate Confidence
Sun et al. [48]	2025	1	3	3	7	Moderate Confidence
Balletshofer et al. [70]	2025	1	3	3	7	Moderate Confidence
Kóczi a Sárosi [69]	2025	3	2	3	8	High Confidence
Palmieri et al. [66]	2025	1	2	3	6	Moderate Confidence
Caneschi et al. [38]	2026	3	3	3	9	High Confidence

## References

[1] Raffik R., Sathya R.R., Vaishali V., Balavedhaa S. Industry 5.0: Enhancing Human-Robot Collaboration Through Collaborative Robots—A Review. Proceedings of the 2023 2nd International Conference on Advancements in Electrical, Electronics, Communication, Computing and Automation (ICAECA).

[2] Yitmen I., Almusaed A. (2024). Synopsis of Industry 5.0 Paradigm for Human-Robot Collaboration. Artificial Intelligence.

[3] Franklin C.S., Dominguez E.G., Fryman J.D., Lewandowski M.L. (2020). Collaborative robotics: New era of human–robot cooperation in the workplace. J. Saf. Res..

[4] Hameed A., Ordys A., Możaryn J., Sibilska-Mroziewicz A. (2023). Control System Design and Methods for Collaborative Robots: Review. Appl. Sci..

[5] Behrens R., Zimmermann J., Wang Z., Herbster S., Elkmann N. (2024). Development of Biomechanical Response Curves for the Calibration of Biofidelic Measuring Devices Used in Robot Collision Testing. J. Biomech. Eng..

[6] Kragic D., Gustafson J., Karaoguz H., Jensfelt P., Krug R. Interactive, Collaborative Robots: Challenges and Opportunities. Proceedings of the Twenty-Seventh International Joint Conference on Artificial Intelligence.

[7] Zimmermann J., Huelke M., Clermont M. (2022). Experimental Comparison of Biofidel Measuring Devices Used for the Validation of Collaborative Robotics Applications. Int. J. Environ. Res. Public Health.

[8] Kirschner R.J., Kirschner J., Abdolshah S., Haddadin S. (2022). ISO/TS 15066: How Different Interpretations Affect Risk Assessment. arXiv.

[9] (2025). Robotics—Safety Requirements—Part 2: Industrial Robot Applications and Robot Cells.

[10] (2016). Robots and Robotic Devices—Collaborative Robots.

[11] (2018). Technical Report–Industrial Robots and Robot Systems–Safety Requirements–Testing Methods for Power & Force Limited Collaborative Applications.

[12] (2023). Robotics—Collaborative Applications—Test Methods for Measuring Forces and Pressures in Human-Robot Contacts.

[13] (2011). Robots and Robotic Devices—Safety Requirements for Industrial Robots—Part 1: Robots.

[14] (2011). Robots and Robotic Devices—Safety Requirements for Industrial Robots—Part 2: Robot Systems and Integration.

[15] (2025). Robotics—Safety Requirements—Part 1: Industrial Robots.

[16] Hartmann D., Hamříková K., Vysocký A., Laciok V., Bernatík A. (2026). Evolution of safety requirements in industrial robotics: Comparative analysis of ISO 10218-1/2 (2011 vs. 2025) and integration of ISO/TS 15066. Results Eng..

[17] (2010). Safety of Machinery—General Principles for Design—Risk Assessment and Risk Reduction.

[18] (2019). Safety of Machinery—Instruction Handbook—General Drafting Principles.

[19] Arents J., Abolins V., Judvaitis J., Vismanis O., Oraby A., Ozols K. (2021). Human–Robot Collaboration Trends and Safety Aspects: A Systematic Review. J. Sens. Actuator Netw..

[20] Valori M., Scibilia A., Fassi I., Saenz J., Behrens R., Herbster S., Bidard C., Lucet E., Magisson A., Schaake L. (2021). Validating Safety in Human–Robot Collaboration: Standards and New Perspectives. Robotics.

[21] Samarathunga S.M.B.P.B., Valori M., Legnani G., Fassi I. (2025). Assessing Safety in Physical Human–Robot Interaction in Industrial Settings: A Systematic Review of Contact Modelling and Impact Measuring Methods. Robotics.

[22] Kitchenham B., Brereton P. (2013). A systematic review of systematic review process research in software engineering. Inf. Softw. Technol..

[23] Kitchenham B., Charters S. (2007). Guidelines for Performing Systematic Literature Reviews in Software Engineering.

[24] Samarathunga S.M.B.P.B., Valori M., Faglia R., Fassi I., Legnani G. (2024). Considerations on the Dynamics of Biofidelic Sensors in the Assessment of Human–Robot Impacts. Machines.

[25] Zhu X., Zhang K., Hua X. (2024). Consistency Analysis and Suggestions of Collision Measurement in Human–Robot Collaboration Safety Evaluation. Int. J. Robot. Autom..

[26] Staab H., Byner C., Clever D., Matthias B. A Pendulum Apparatus to Evaluate Unconstrained Human-Robot Contact. Proceedings of the 52nd International Symposium on Robotics (ISR 2020).

[27] Scibilia A., Valori M., Pedrocchi N., Fassi I., Herbster S., Behrens R., Saenz J., Magisson A., Bidard C., Kuhnrich M. (2021). Analysis of Interlaboratory Safety Related Tests in Power and Force Limited Collaborative Robots. IEEE Access.

[28] Zimmermann J., Clermont M., Nischalke-Fehn G. (2025). Excerpt from a Practical Risk Assessment of the Hand-Arm Region for Workplaces with Collaborative Robots. Springer Proceedings in Advanced Robotics.

[29] D’Antona A., Farsoni S., Rizzi J., Bonfè M. (2025). A Variable Stiffness System for Impact Analysis in Collaborative Robotics Applications with FPGA-Based Force and Pressure Data Acquisition. Sensors.

[30] Fischer C., Steiner M., Neuhold M., Papa M., Markis A., Schlund S. (2022). An Investigation of the Measurement of Transient Contacts in Human-Robot Interaction. Mechanisms and Machine Science.

[31] Schneider C., Seizmeir M.M., Suchanek T., Hutter-Mironovová M., Bdiwi M., Putz M. Empirical Analysis of the Impact of Additional Padding on the Collaborative Robot Velocity Behavior in Transient Contact Cases. Proceedings of the 18th International Conference on Informatics in Control, Automation and Robotics (ICINCO).

[32] Schneider C., Suchanek T., Hutter-Mironovová M., Bdiwi M., Putz M. (2023). Approximation Methods and Reference Values for Maximum Allowed Collaborative Operating Speeds in Quasi-Static and Transient Contact Cases. Lecture Notes in Electrical Engineering.

[33] Schlotzhauer A., Stotz T., Awad R., Kraus W. (2022). Virtual Validation of Power and Force Limiting Setups in Human-Robot-Collaboration. Procedia CIRP.

[34] Hüsing E., Cañari J., Corves B. (2023). Rounded Edges and Chamfers as a Protective Measure in Quasi-Static Contact Events. Mechanisms and Machine Science.

[35] Cordero C.A., Carbone G., Ceccarelli M., Echávarri J., Muñoz J.L. (2014). Experimental tests in human–robot collision evaluation and characterization of a new safety index for robot operation. Mech. Mach. Theory.

[36] Rajaei N., Fujikawa T., Yamada Y. Experimental Investigation of Human Soft Tissue Behavior for Constructing Human-Robot Contact Force-Displacement Measuring System. Proceedings of the 2021 IEEE International Conference on Intelligence and Safety for Robotics (ISR).

[37] Dagalakis N.G., Yoo J.-M., Oeste T. (2016). Human-robot collaboration dynamic impact testing and calibration instrument for disposable robot safety artifacts. Ind. Robot.

[38] Caneschi A., Bottin M., Rosati G. (2026). Modeling Human–Robot Impact Dynamics in Collaborative Applications. Actuators.

[39] Hirata A., Shimaoka Y., Okamoto T., Watanabe R., Yamada Y. Evaluation of Biofidelity and a Proposal for Simplification of a Human-inspired Safety Dummy. Proceedings of the 2021 IEEE International Conference on Intelligence and Safety for Robotics (ISR).

[40] Iki Y., Yamada Y., Akiyama Y., Okamoto S., Liu J. Designing A Dummy Skin by Evaluating Contacts between A Human Hand and A Robot End Tip. Proceedings of the 2020 IEEE/RSJ International Conference on Intelligent Robots and Systems (IROS).

[41] Fischer C., Neuhold M., Steiner M., Haspl T., Rathmair M., Schlund S. (2023). Collision Tests in Human-Robot Collaboration: Experiments on the Influence of Additional Impact Parameters on Safety. IEEE Access.

[42] Liu J., Yamada Y., Akiyama Y., Okamoto S., Iki Y. (2023). Development of Dummy Based on Impedance Properties of Human Soft Tissue Using a Nonlinear Viscoelastic Model. IEEE Access.

[43] Svarny P., Rozlivek J., Rustler L., Hoffmann M. 3D Collision-Force-Map for Safe Human-Robot Collaboration. Proceedings of the 2021 IEEE International Conference on Robotics and Automation (ICRA).

[44] Matthias B., Oberer-Treitz S., Ding H. Experimental Characterization of Collaborative Robot Collisions. Proceedings of the 41st International Symposium on Robotics (ISR/Robotik 2014).

[45] Kim J., Alspach A., Yamane K. 3D printed soft skin for safe human-robot interaction. Proceedings of the 2015 IEEE/RSJ International Conference on Intelligent Robots and Systems (IROS).

[46] Kirschner R.J., Jantalia J., Mansfeld N., Abdolshah S., Haddadin S. CSM: Contact Sensitivity Maps for Benchmarking Robot Collision Handling Systems. Proceedings of the 2021 IEEE International Conference on Robotics and Automation (ICRA).

[47] Rustler L., Misar M., Hoffmann M. Adaptive Electronic Skin Sensitivity for Safe Human-Robot Interaction. Proceedings of the 2024 IEEE-RAS 23rd International Conference on Humanoid Robots (Humanoids).

[48] Sun X., Zhou L., Ren Z., Chen B., Li Y. Multidimensional Impact Characterization in Collaborative Robotics: A Gazebo-Based Collision Testing System. Proceedings of the 2025 IEEE International Conference on Mechatronics and Automation (ICMA).

[49] Herbster S., Behrens R., Elkmann N. (2023). Modeling the Contact Force in Constrained Human–Robot Collisions. Machines.

[50] Hornung L., Sóti G., Wurll C. (2024). A Step Towards a Finite Element Model for an Impact Situation in Human-Robot Interaction. Lecture Notes in Networks and Systems.

[51] Clever D., Byner C., Staab H., Matthias B. On Peak and Integral Criteria to Assess Physical Contact in Human-Robot-Collaboration (HRC). Proceedings of the 54th International Symposium on Robotics (ISR Europe 2022).

[52] Case J.C., Rangarajan N., Falco J., Kimble K. Towards the Development of Soft Force and Pressure Sensors for Robot Safety Applications. Proceedings of the 2021 IEEE Sensors.

[53] Povse B., Haddadin S., Belder R., Koritnik D., Bajd T. (2015). A tool for the evaluation of human lower arm injury: Approach, experimental validation and application to safe robotics. Robotica.

[54] Samarathunga S.M.B.P.B., Bertagna A., Fassi I., Valori M., Pagani R., Vetturi D., Legnani G. A Pendulum Approach to Understanding the Dynamics of Transient Contact in Human-Robot Collaboration. Proceedings of the 20th IEEE/ASME International Conference on Mechatronic and Embedded Systems and Applications (MESA).

[55] Wang A., Zheng J., Zheng H., Lin D. Research on test method of collaborative robot safety. Proceedings of the 49th International Symposium on Robotics (ISR 2017).

[56] Lachner J., Allmendinger F., Hobert E., Hogan N., Stramigioli S. (2021). Energy budgets for coordinate invariant robot control in physical human–robot interaction. Int. J. Rob. Res..

[57] Byner C., Clever D., Staab H., Matthias B. An extended two-mass model for clamping hazards in human-robot-collaboration: Peak forces and permissible speeds. Proceedings of the 54th International Symposium on Robotics (ISR Europe 2022).

[58] Kovinčić N., Gattringer H., Müller A., Brandstötter M. Physics Guided Machine Learning Approach to Safe Quasi-Static Impact Situations in Human-Robot Collaboration Following the Power and Force Limiting Method of the ISO/TS 15066 Standard. Proceedings of the 19th International Conference on Multibody Systems, Nonlinear Dynamics, and Control (MSNDC).

[59] Kovinčić N., Gattringer H., Müller A., Brandstötter M. (2024). Physics-Guided Machine Learning Approach to Safe Quasi-Static Impact Situations in Human–Robot Collaboration. J. Comput. Nonlinear Dyn..

[60] Herbster S., Behrens R., Elkmann N. (2021). A New Conversion Method to Evaluate the Hazard Potential of Collaborative Robots in Free Collisions. Springer Proceedings in Advanced Robotics.

[61] Han D., Park M.Y., Choi J., Shin H., Behrens R., Rhim S. (2024). Evaluation of force pain thresholds to ensure collision safety in worker-robot collaborative operations. Front. Robot. AI.

[62] Shin H., Kim S., Seo K., Rhim S. A Real-Time Human-Robot Collision Safety Evaluation Method for Collaborative Robot. Proceedings of the 3rd IEEE International Conference on Robotic Computing (IRC).

[63] Kirschner R.J., Mansfeld N., Abdolshah S., Haddadin S. Experimental Analysis of Impact Forces in Constrained Collisions According to ISO/TS 15066. Proceedings of the 2021 IEEE International Conference on Intelligence and Safety for Robotics (ISR).

[64] Ganglbauer M., Ikeda M., Plasch M., Pichler A. (2020). Human in the loop online estimation of robotic speed limits for safe human robot collaboration. Procedia Manuf..

[65] Povse B., Koritnik D., Bajd T., Munih M. Mechanical model of human lower arm. Proceedings of the 7th Annual ACM/IEEE International Conference on Human-Robot Interaction (HRI).

[66] Palmieri G., Ponzetti S., Scoccia C., Costa D., Zollo L. Dynamic Characterization of Biofidelic Sensors for Human-Robot Impact Assessment. Proceedings of the 21st IEEE/ASME International Conference on Mechatronic and Embedded Systems and Applications (MESA).

[67] St-Jean A., Dorval F., Plante J.-S., Lussier-Desbiens A. (2024). Magnetorheological-Actuators: An Enabling Technology for Fast, Safe, and Practical Collaborative Robots. IEEE Trans. Robot..

[68] Ponikelský J., Černohlávek V., Štěrba J., Houška P. (2023). Research of Robots in Cooperative Mode in Human Body Part Detection. Manuf. Technol..

[69] Kóczi D., Sárosi J. (2025). Analysis of Collision Types in Collaborative Robots Using Mechanism Actuated by Pneumatic Artificial Muscle. Actuators.

[70] Balletshofer J., Kirschner R.J., Althoff M. Collision Mass Map for Safe and Efficient Human-Robot Interaction. Proceedings of the 2025 IEEE/RSJ International Conference on Intelligent Robots and Systems (IROS).

[71] Steinecker T., Kurdas A., Mansfeld N., Hamad M., Kirschner R.J., Abdolshah S., Haddadin S. Mean Reflected Mass: A Physically Interpretable Metric for Safety Assessment and Posture Optimization in Human-Robot Interaction. Proceedings of the 2022 International Conference on Robotics and Automation (ICRA).

[72] Schlotzhauer A., Kaiser L., Wachter J., Brandstotter M., Hofbaur M. On the trustability of the safety measures of collaborative robots: 2D Collision-force-map of a sensitive manipulator for safe HRC. Proceedings of the 2019 IEEE 15th International Conference on Automation Science and Engineering (CASE).

[73] Huang S., Gao M., Liu L., Chen J., Zhang J. (2022). Collision Detection for Cobots: A Back-Input Compensation Approach. IEEE/ASME Trans. Mechatron..

[74] Kirschner R.J., Kurdas A., Karacan K., Junge P., Birjandi S.A.B., Mansfeld N., Abdolshah S., Haddadin S. Towards a Reference Framework for Tactile Robot Performance and Safety Benchmarking. Proceedings of the 2021 IEEE/RSJ International Conference on Intelligent Robots and Systems (IROS).

[75] Svarny P., Rozlivek J., Rustler L., Sramek M., Deli Ö., Zillich M., Hoffmann M. (2022). Effect of active and passive protective soft skins on collision forces in human–robot collaboration. Rob. Comput. Integr. Manuf..

[76] Rosenstrauch M.J., Kruger J. Safe human-robot-collaboration-introduction and experiment using ISO/TS 15066. Proceedings of the 2017 3rd International Conference on Control, Automation and Robotics (ICCAR).

[77] Heng W., Yang G., Pang G., Ye Z., Lv H., Du J., Zhao G., Pang Z. (2020). Fluid-Driven Soft CoboSkin for Safer Human–Robot Collaboration: Fabrication and Adaptation. Adv. Intell. Syst..

[78] Dombrowski U., Stefanak T., Reimer A. (2018). Simulation of human-robot collaboration by means of power and force limiting. Procedia Manuf..

[79] Ponikelský J., Chalupa M., Černohlávek V., Štěrba J. (2024). Force and Pressure Dependent Asymmetric Workspace Research of a Collaborative Robot and Human. Symmetry.

[80] Li F. Evaluation of a Finger Dummy with a Built-in Sensor System for Safety Assessment. Proceedings of the 2024 IEEE/SICE International Symposium on System Integration (SII).

[81] Nguyen V., Case J. (2022). Compensation of electrical current drift in human–robot collision. Int. J. Adv. Manuf. Technol..

[82] Emiliani F., Bajrami A., Costa D., Palmieri G., Polucci D., Leoni C., Callegari M. (2024). Design and Prototyping of a Collaborative Station for Machine Parts Assembly. Machines.

[83] Jeanneau G., Bégoc V., Briot S. (2023). A Reduced Mass-Spring-Mass-Model of Compliant Robots Dedicated to the Evaluation of Impact Forces. J. Mech. Robot..

[84] Park M.Y., Han D., Lim J.H., Shin M.K., Han Y.R., Kim D.H., Rhim S., Kim K.S. (2019). Assessment of pressure pain thresholds in collisions with collaborative robots. PLoS ONE.

[85] Fischer C., Gregshammer F., Steiner M., Neuhold M., Schlund S. (2024). Personalized Safety: Considering the Worker’s Anthropometry in Safety Evaluation of Human-Robot Collaboration. Springer Proceedings in Advanced Robotics.

[86] Zurlo D., Heitmann T., Morlock M., De Luca A. Collision Detection and Contact Point Estimation Using Virtual Joint Torque Sensing Applied to a Cobot. Proceedings of the 2023 IEEE International Conference on Robotics and Automation (ICRA).

[87] Mujica M., Benoussaad M., Fourquet J.-Y. Simulated Framework for Physical Human-Robot Collaboration to Co-Manipulate Objects. Proceedings of the 17th International Conference on Control, Automation, Robotics and Vision (ICARCV).

[88] Suita K., Okawa Y. (2025). Practical evaluation and consideration of viscoelastic covering materials based on simple contact detection for human robot collaboration and harmonization. Adv. Robot..

[89] Geiger L., Guadarrama-Olvera J.R., Cheng G. Normative Safety Regulations for Collaborative Robots. Proceedings of the 2025 IEEE/SICE International Symposium on System Integration (SII).

[90] (2023). Safety of Machinery—Safety-Related Parts Of Control Systems—Part 1: General Principles for Design.

[91] Lucci N., Lacevic B., Zanchettin A.M., Rocco P. (2020). Combining Speed and Separation Monitoring With Power and Force Limiting for Safe Collaborative Robotics Applications. IEEE Robot. Autom. Lett..

[92] Manzardo M., Vidoni R. (2025). On the improvement of the combination of Power and Force Limiting and Speed and Separation Monitoring for an effective Human Robot Collaboration. Procedia Comput. Sci..

[93] Niquet K., Patel N., Schrödel F., Jahn M., Varelmann S. Prototype of an intelligent textile based robot skin to expand potential applications for cobot’s. Proceedings of the 2023 8th International Conference on Mechanical Engineering and Robotics Research (ICMERR).

[94] Zhou Y., Zhao J., Lu P., Wang Z., He B. (2024). TacSuit: A Wearable Large-Area, Bioinspired Multimodal Tactile Skin for Collaborative Robots. IEEE Trans. Ind. Electron..

[95] Tsuji S., Kohama T. (2020). Self-Capacitance Proximity and Tactile Skin Sensor With Shock-Absorbing Structure for a Collaborative Robot. IEEE Sens. J..

[96] Calcagni M.T., Scoccia C., Battista G., Palmieri G., Palpacelli M. Collaborative Robot Sensorization with 3D Depth Measurement System for Collision Avoidance. Proceedings of the 2022 18th IEEE/ASME International Conference on Mechatronic and Embedded Systems and Applications (MESA).

[97] Wang C., Wei C., Bai S., Li Y., Tian X., Zhou L. HCRI: A ROS2 Human Collision Object Interface for Robotic Manipulation Planning. Proceedings of the 2023 IEEE International Conference on Real-time Computing and Robotics (RCAR).

[98] Scimmi L.S., Melchiorre M., Mauro S., Pastorelli S.P. Implementing a Vision-Based Collision Avoidance Algorithm on a UR3 Robot. Proceedings of the 2019 23rd International Conference on Mechatronics Technology (ICMT).

[99] Cheng Y., Tomizuka M. (2022). Long-Term Trajectory Prediction of the Human Hand and Duration Estimation of the Human Action. IEEE Robot. Autom. Lett..

[100] Liu S., Zhang J., Wang L., Gao R.X. (2024). Vision AI-based human-robot collaborative assembly driven by autonomous robots. CIRP Ann..

[101] Makris S., Aivaliotis P. (2022). AI-based vision system for collision detection in HRC applications. Procedia CIRP.

[102] Gruenefeld U., Prädel L., Illing J., Stratmann T., Drolshagen S., Pfingsthorn M. Mind the ARm: Realtime visualization of robot motion intent in head-mounted augmented reality. Proceedings of the Conference on Mensch und Computer.

[103] Ong S.K., Yew A.W.W., Thanigaivel N.K., Nee A.Y.C. (2020). Augmented reality-assisted robot programming system for industrial applications. Rob. Comput. Integr. Manuf..

[104] Subramanian K., Arora S., Adamides O., Sahin F. Using Mixed Reality for Safe Physical Human-Robot Interaction. Proceedings of the 2024 IEEE Conference on Telepresence.

[105] Paniti I., Nacsa J., Kovács P., Szűr D. VR and Depth Camera based Human-Robot Collision Predictor System with 3-Finger Gripper Assisted Assembly Device. Proceedings of the 2020 23rd International Symposium on Measurement and Control in Robotics (ISMCR).

[106] Behrens R., Pliske G., Umbreit M., Piatek S., Walcher F., Elkmann N. (2022). A Statistical Model to Determine Biomechanical Limits for Physically Safe Interactions With Collaborative Robots. Front. Robot. AI.

[107] Pollak A., Paliga M., Pulopulos M.M., Kozusznik B., Kozusznik M.W. (2020). Stress in manual and autonomous modes of collaboration with a cobot. Comput. Hum. Behav..

[108] Liu B., Fu W., Wang W., Li R., Gao Z., Peng L., Du H. (2022). Cobot Motion Planning Algorithm for Ensuring Human Safety Based on Behavioral Dynamics. Sensors.

[109] Campagna G., Chrysostomou D., Rehm M. Analysis of Facial Features for Trust Evaluation in Industrial Human-Robot Collaboration. Proceedings of the 2024 IEEE International Conference on Advanced Robotics and Its Social Impacts (ARSO).

